# New fossils from Kromdraai and Drimolen, South Africa, and their distinctiveness among *Paranthropus robustus*

**DOI:** 10.1038/s41598-022-18223-7

**Published:** 2022-08-17

**Authors:** José Braga, G. Chinamatira, B. Zipfel, V. Zimmer

**Affiliations:** 1 Centre for Anthropobiology and Genomics of Toulouse, CNRS UMR 5288, Université de Toulouse, Université Paul Sabatier, 37 Allées Jules Guesde, Toulouse, France; 2 Evolutionary Studies Institute, University of the Witwatersrand, PO WITS, Johannesburg, 2050 South Africa; 3 Faculty of Informatics, Technical University of Munich, Munich, Germany

**Keywords:** Classification and taxonomy, Anthropology, Palaeontology, Taxonomy

## Abstract

Most fossil hominin species are sampled with spatial, temporal or anatomical biases that can hinder assessments of their paleodiversity, and may not yield genuine evolutionary signals. We use new fossils from the Kromdraai (Unit P) and Drimolen sites (South Africa) to provide insights into the paleodiversity of the Lower Pleistocene robust australopith, *Paranthropus robustus.* Our focus is the morphology of the temporal bone and the relationships between size and shape (allometry) of the semi-circular canals (SCC), an aspect that has not yet been investigated among southern African australopiths. We find significant size and shape SCC differences between *P. robustus* from Kromdraai, Drimolen and Swartkrans. This site-related variation is consistent with other differences observed on the temporal bone. *P. robustus* from Kromdraai Unit P is distinctive because of its smaller temporal bone and SCC, and its proportionally less developed posterior SCC, independently of age and sex. We emphasize the importance of allometry to interpret paleodiversity in *P. robustus* as either the consequence of differences in body size, or as yet unknown factors. Some features of the inner ear of *P. robustus* represent directional selection soon after its origin, whereas the size and shape variations described here may result from evolutionary changes.

## Introduction

**Figure 1:**
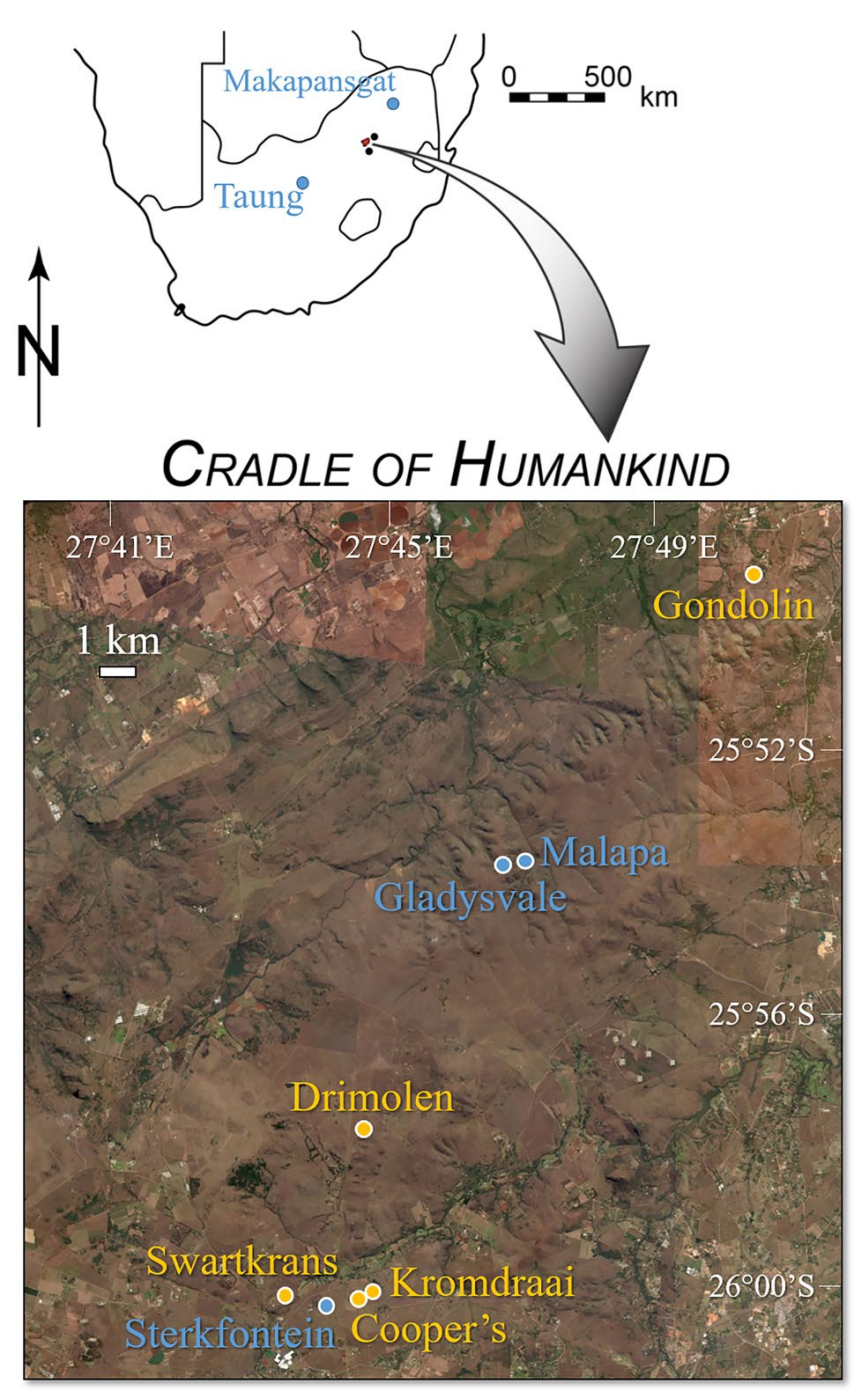
Map showing the location of the five *Australopithecus*-bearing (blue) and the five *Paranthropus robustus*-bearing (yellow) sites in South Africa. Figure created by JB and map generated by using ‘Earth Explorer’, a free in-browser platform to access Landsat satellite imagery (https://earthexplorer.usgs.gov/).

The assessments of morphological variation within early hominin species influence taxonomic and phylogenetic interpretations, and need constant reevaluation when new fossils and analytical methods are available. After the discovery of the type specimen of *Paranthropus robustus* from the site of Kromdraai (Gauteng, South Africa)^1^ (Fig. 1), the taxonomic validity of this Lower Pleistocene robust australopith species was questioned^2^. The view of a variable *P. robustus* species subsequently became conventional when additional discoveries were made at the sites of Kromdraai^3,4^, Swartkrans^5^ and Drimolen^6–8^, with much less evidence being available from the nearby sites of Cooper’s Cave, Gondolin and Sterkfontein (Fig. 1). Morphological variations within *P. robustus* were nevertheless interpreted diversely according to the trait investigated. For instance, the study of the part of the inner ear involved in hearing (the cochlea) provided evidence for directional selection in this species soon after its origin^4^, whereas other variations in cranial morphology were considered as possible evidence for an evolutionary lineage (i.e. an ancestral-descendant sequence of populations) within *P. robustus*^3,7^. Debates regarding the phylogeny of *P. robustus* also began soon after its discovery^1,2^, and have continued ever since^8,9^. In this regard, *P. robustus* has been considered to lie very close to the last common ancestor of the robust australopiths from southern and eastern Africa (i.e., *Paranthropus*)^8^, and to *Australopithecus africanus*^10^ from the Pliocene of southern Africa^9^. Therefore, it is particularly important to investigate whether some fossils from Kromdraai, Drimolen or Swartkrans might represent a more primitive (plesiomorphic) condition for *P. robustus*, with more similarities with the geologically older *A. africanus*.

In this study, we expand the knowledge of the paleodiversity patterns within *P. robustus* by contributing new fossil evidence from the sites of Drimolen and Kromdraai (Table 1, Text S1). In doing so, our main goal is to test whether and how we can identify distinct *P. robustus* groups consistently with their provenience from the sites of Kromdraai, Drimolen and Swartkrans, that are enclosed within an area of approximately 10 km^2^ (Fig. 1). The geographically constrained morphological variation measured among three main *P. robustus* samples is compared with differences observed in other southern African early hominins, including *A. africanus*. To achieve this, we study the outer and inner morphology of the temporal bone, including digital endocasts of the inner ear bony capsule (or bony labyrinth, BL) reconstructed from micro-computed tomography (micro-CT). For the first time, we use this approach to investigate the type specimen of *A. africanus* from Taung^10^ that has attracted particular attention in the context of the origin of southern and eastern African *Paranthropus*^9^. We also newly use approaches well suited for the study of biological scaling, i.e. the description of shape changes with size (allometry)^11^ and comparisons of shapes^4,12^.

**Table 1:** List of the fossil hominin specimens investigated in this study.

Specimen	Site/Stratigraphic unit	Preservation/age	References
***Paranthropus robustus*, Kromdraai**			
TM 1517	Younger than Units QRP	Partial cranium w/teeth	^1^
KB 6067	Units QR (“Member 3” *)	Isolated petrous bone	^3^
KW 9600	Unit P (“Member 2” *)	Partial cranium	^4^
KW 9700	Unit P (“Member 2” *)	Isolated petrous bone	^4^
KW 9900	Unit P (“Member 2” *)	Cranial fragments w/teeth	This study
KW 10840	Unit P (“Member 2” *)	Partial cranium w/teeth	^4^
***Paranthropus robustus*, Drimolen**			
DNH 22	Drimolen Main Quarry	Cranial fragments w/teeth	^6^
DNH 34	Drimolen Main Quarry	Cranial fragments	This study
DNH 60	Drimolen Main Quarry	Cranial fragments w/teeth	This study
***Paranthropus robustus*, Swartkrans**			
SK 879	Member 1	Isolated petrous bone	^3^
SKW 18/SK 52	Member 1	Partial cranium w/teeth	^2^
SK 83	Member 1	Partial cranium w/teeth	^2^
***Australopithecus africanus***,** Makapansgat**			
MLD 31	Unknown (mine dumps)	Isolated petrous bone	^13^
***Australopithecus africanus*, Sterkfontein**			
Sts 5	Member 4	Cranium	^14^
Sts 19	Member 4	Partial cranium	^14^
Stw 98	Member 4	Partial temporal bone	^15^
Stw 252/255^§^	Member 4	Partial cranium w/teeth	^15^
Stw 329	Member 4	Partial temporal bone	^15^
Stw 498e^§^	Member 4	Partial cranium w/teeth	^3^
Stw 504/505^§^	Member 4	Partial cranium w/teeth	^16^
Stw 573^§^	Member 2	Partial skeleton w/cranium	^17^
Stw 578^§^	Jacovec Cavern	Partial cranium w/teeth	^18^
***Australopithecus africanus*, Taung**			
Taung	Taung Quarry	Cranium w/teeth	^9^
**Early *Homo***			
SK 847	Swartkrans/Member 1	Partial cranium w/teeth	^19^
**Indeterminate, Sterkfontein**			
StW 151	Member 5 ?	Partial cranium w/teeth	^20^
StW 53	Member 5A	Partial cranium w/teeth	^17,19^

*This terminology was used in references 18 and 19.

^§^Specimen that has been considered to represent a species of *Australopithecus* with robust affinities.

?means that the stratigraphic provenience of this specimen is uncertain.

**Figure 2:**
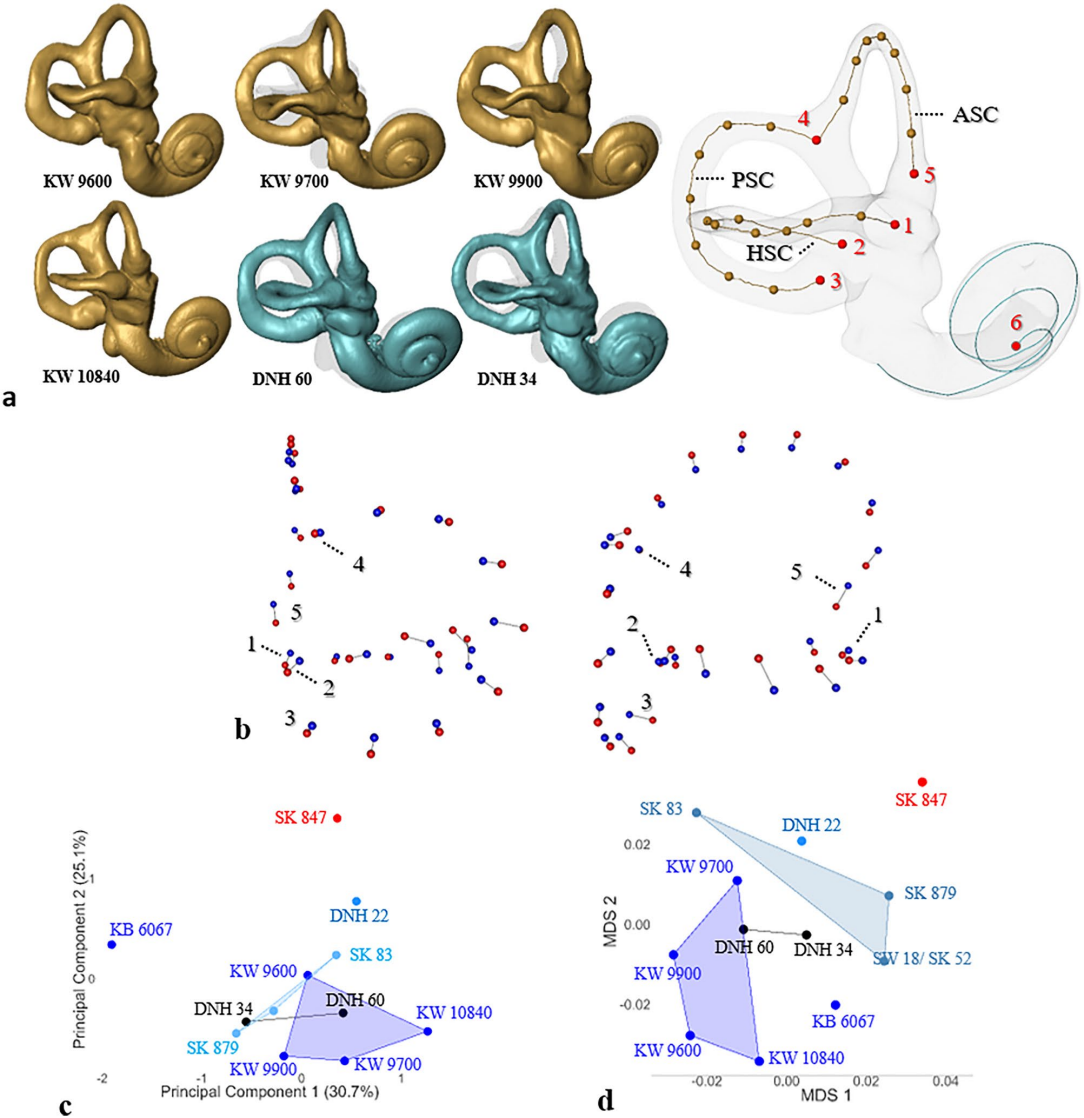
Bony labyrinths (BL) of six new *Paranthropus robustus* specimens and binary plots of Principal Component Analyses (PCA) showing their closest neighbours. (**a**) BLs from Kromdraai Unit P (in gold, KW 9600, KW 9700, KW 9900 and KW 10840) and Drimolen Main Quarry (in blue, DNH 34 and DNH 60) shown in lateral view with same scales and orientations (all aligned to KW 10840), and 3D model shown in transparency and in lateral view to illustrate the cochlear curve (in blue), the three centerlines of the horizontal, posterior and anterior semicircular canals (SCC) (HSC, PSC and ASC, respectively), six landmarks (Ld) (in red) and semilandmarks (in gold). Five landmarks are placed at fixed locations, at the extremities of SCCs. The centerlines are expressed as 7 semilandmarks per SCC. Ld.1, center of the ampulla of HSC; Ld.2, posterolateralmost point of the HCC centerline; Ld.3, center of the ampulla of the PSC; Ld.4, bifurcation point of the common crus; Ld.5, center of the ampulla of the ASC. c,d, Binary plots from a PCA after a Generalized Procrustes Analysis (GPA) (**c**), and from computational anatomy (with multidimensional scaling) (**d**) of the BL in *Paranthropus robustus* from the Kromdraai (n = 5, dark blue), Drimolen (DNH 22) and Swartkrans (n = 2) (light blue) sites, one early *Homo* specimen from Swartkrans (SK 847, red), and two additional specimens from Drimolen (DNH 34 and DNH 60, black) here considered as “indeterminate” and projected onto the statistical space to identify their closest neighbours (“Methods”). (**b**) Vectors showing the differences between the configurations of landmarks and semilandmarks of the minimum (blue) and maximum (red) values along PC2 in the shape space illustrated in (**c**).

Here, with new methods and descriptions of new fossils from Kromdraai and Drimolen (Table 1, Fig. 2a, Text S1, Figs S1–6, Tables S1–2), we address the following limitations of previous assessments of paleodiversity within *P. robustus*. First, the *P. robustus* sample has been biased toward adult cranial remains from the sites of Swartkrans and Drimolen, with the notable absence of any such evidence from Kromdraai. Second, the morphological comparisons between the three main *P. robustus*-bearing sites of Swartkrans, Drimolen and Kromdraai have been focused on simple dental metrics^5,6^, with more limited evidence from the latter site. Third, even though the influence of size on shape diversification across species (evolutionary allometry) are widely accepted^21^, they remain unexplored among *P. robustus* and other southern African australopiths. It is therefore important to investigate such effects. Fourth, the size differences between the smaller and the larger craniodental *P. robustus* specimens are most often interpreted as sexual dimorphism^22^. While facial and dental size is commonly used to discuss sexual dimorphism in body size among *P. robustus*, variation in size of other cranial traits remains underexplored towards this end. Fifth, the size distribution of *P. robustus* craniodental remains is usually considered as skewed due to sampling biases (i.e., processes of accumulation of fossil assemblages that preclude paleodemographic reconstructions)^22,23^. The full extent of size variation among *P. robustus* is therefore considered as underestimated^23^. Finally, it is worth noting that the *P. robustus*-bearing sites represent uncorrelated depositional sequences and their age-range estimates overlap extensively with one another^8^, which entails within-lineage evolutionary changes difficult to evaluate.

The importance of reporting on new *P. robustus* discoveries from Kromdraai is both that they add the first fully adult specimen from this site (KW 9900) (Table 1, Text S1, Figs S2–5, Tables S1–2) and that they represent the first sample of this species from Unit P, located in the earliest part of the stratigraphic sequence (Table 1)^4,24–26^. Unit P was previously referred to as “Member 2”^27–29^, and was considered as “sterile”^27^ prior to the commencement of new excavations at Kromdraai in 2014^28–30^. The new *P. robustus* assemblage from Kromdraai Unit P (Table 1) is older than other conspecific specimens recovered from this site before 2014 (including the holotype of this species, TM 1517^1^, and the KB 6067 specimen from the younger Unit Q-R^3,25,26^). Paleomagnetism and biochronology suggest that both Unit P and Units Q-R are older than 1.95 Ma, whereas the *P. robustus* type specimen from Kromdraai (TM 1517)^1^ might be younger^3,25,26^. Therefore, the Kromdraai *P. robustus* specimens represent a series of temporally successive samples that potentially record evolutionary changes.

The use of new fossil evidence from Kromdraai and Drimolen also has implications to discuss the phylogenetic relationships between *P. robustus* and *A. africanus*. Two hypotheses are important to consider regarding this particular point. First, *A. africanus* is usually considered as phylogenetically basal to robust australopiths from southern and eastern Africa (i.e., *Paranthropus*) and the genus *Homo*^29^. Second, it has long been considered that robust (i.e., *Paranthropus*-like) features may be present in *A. africanus* from the southern African sites of Makapansgat^30^, Taung^9^ and Sterkfontein^17,15^ (Fig. 1). However, there are only a few dental^31,32^, facial^33^ and basicranial^34,19^ features linking *P. robustus* and *A. africanus*, to the exclusion of other australopiths. It also remains unclear whether australopith remains from Sterkfontein and Makapansgat represent a single species (i.e., *A. africanus*)^4,31^ or include a second taxon with purported robust australopith affinities^17^.

In this study, we investigate features of the temporal bone that have been considered of some taxonomic value in discussing robust affinities in *A. africanus*^15^, and to suggest the ancestral status and/or the smaller size of some *P. robustus* specimens from either Kromdraai^3^ or Drimolen^8^. Following a recent study of cochlear shape among southern African fossil hominins^4^, we investigate the shape correlates of semi-circular canal (SCC) size differences among *P. robustus* and *A. africanus* (evolutionary allometry) (Fig. 2a). Variation in BL morphology related to allometry is ubiquitous among primates^35^ but has not yet been investigated in *A. africanus* and *P. robustus*. We therefore newly add evolutionary allometry to approaches of BL that already proved their utility to reveal species-specific characters among primates^13,36,37^ and other mammals^38,39^, and differences between *A. africanus* and *P. robustus* that exceed those between modern humans, chimpanzees and gorillas^4^.

We investigate a sample of southern African early hominins (n = 25) (Table 1, Table S1), including two undescribed fossils recovered from Drimolen in the late 1990s (DNH 34 and DH 60)^40^ and four *P. robustus* specimens from Kromdraai Unit P^4^ (Table 1, Fig. 2a, “Methods”, Text S1, Figs S1–6, Table S1). The sample from Kromdraai Unit P includes the first nearly complete temporal bone of a *P. robustus* child known (KW 10840) (Fig. S6). The only undisputed early *Homo* specimen represented in our sample (SK 847)^19^ was recovered from Swartkrans Member 1 “Hanging Remnant”. We also discuss whether our results help to resolve the taxonomic assignation of two specimens from Sterkfontein here considered as “indeterminate”: StW 53 from Member 5A, and StW 151 with less certain stratigraphic provenience (“Methods”, Text S1). Finally, we add four samples (n = 10, for each one) representing modern humans (*Homo sapiens*), bonobos (*Pan paniscus*), common chimpanzees (*P. troglodytes*) and gorillas (*Gorilla gorilla*) (Table S1), in order to discuss the potential effects of sexual dimorphism and evolutionary allometry on the paleodiversity observed within the fossil sample.

## Results

### The temporal bone

We first examine features on the outer aspect of the temporal bone that have been used to describe an ancestral-descendant sequence among robust australopiths, and particularly *P. robustus*^8,41^. The right temporal bone of KW 9900 represents the first fully adult *P. robustus* cranial remain from Kromdraai (Table 1, Text S1, Figs S3–S4). It was found in association with the right malleus and a heavily worn maxillary dentition (Fig. S5, Table S2). The overall size of the KW 9900 temporal bone is notably smaller than in the geologically younger *P. robustus* holotype (TM 1517) from Kromdraai (Fig. S3), and any conspecifics from Swartkrans. In KW 9000, the length of the tympanic bone (22.1 mm) and the area the external auditory meatus (88.4 mm^2^) are the smallest among *P. robustus*. In KW 9000, the degree of inferior projection of the postglenoid process relative to the zygomatic root (12.5 mm) is reduced when compared to other *P. robustus *specimens, including DNH 7, a presumed female from Drimolen (Table 1, Text S1). The morphology of the KW 9900 temporal bone also departs from the pattern yet described for *P. robustus* in two important features (Text S1). First, in lateral view, the most inferior point on the preserved remnant of the mastoid process (or mastoid tip) projects anteroposteriorly below the external auditory meatus (Fig. S4). This reflects an anteriorly positioned mastoid tip in relation to the asterion-porion distance. Our minimum estimate of this distance yields a “mastoid tip position index”^41^ not higher than 30%, which is lower than that of the two other measurable *P. robustus* crania from Drimolen (DNH 7) and Swartkrans Member 1 (SKW 18/SK 52), and more similar to the condition in *A. africanus* from Makapansgat (MLD 37/38) and *A. afarensis* (A.L. 444-2)^8^ (Text S1). Second, unlike in the *P. robustus* type specimen (TM 1517) and fossils from Swartkrans, the relatively thin inferolateral edge of the KW 9900 tympanic bone (2.7 mm) is slightly recessed from the lateral margin of the mastoid process and the suprameatal crest, with no peculiar lateral extension at this level (Figs. S3–4). Moreover, when seen in inferior view (Fig. S4), KW 9900 does not evince a widened and flared lateral portion of its tympanic bone (or “trumpet” shape), a feature that has been considered as typical of *P. robustus*^42^.

### Taxonomic attribution of DNH 34 and DNH 60 from Drimolen

The DNH 34 and DH 60 specimens represent two undescribed fossils recovered from Drimolen in the late 1990s^40^. It is therefore important to determine their taxonomic attribution. The fossil assemblage from Drimolen contains hominin specimens representing only *P. robustus*, early *Homo* and *Homo aff. erectus*^6–8^. Accordingly, we discuss the taxonomic attribution of DNH 34 and DNH 60 (Table 1, Text S1) by using a comparative sample representing only *P. robustus* and *Homo* (but not *A. africanus*) (Fig. 2c,d). We also use a sample of modern *H. sapiens* because the early *Homo* specimen SK 847 from Swartkrans has been found to be very similar in SCC morphology^3^. In doing so, we use two distinct Principal Component Analyses (PCA) (Fig. 2c, Fig. S7) and one multidimensional scaling (MDS) (Fig. 2d) obtained from computational anatomy^4,12^ (“Methods”). We project DNH 34 and DNH 60 onto the two PC1/PC2 (Fig. 2c, Fig. S7) and the MDS1/MDS2 (Fig. 2d) representations, because we initially consider them “indeterminate” and we aim to identify their closest neighbours.

The first PCA (Fig. 2c) is applied after a Generalized Procrustes Analysis (GPA) of 5 landmarks and 7 semilandmarks per SCC (Fig. 2a) (“Methods”). The second PCA (Fig. S7) is obtained from 12 indices and 10 angles of the BL (“Methods”, Table S3). As detailed in previous studies of dental surfaces^12^ and cochlear shapes^4^, computational anatomy uses deformation-based methods in a non-linear geometric framework to define size-independent differences between the SCCs that are illustrated by the MDS. All the three analyses (the two PCAs and the MDS) unambiguously associate both DNH 34 and DNH 60 with *P. robustus* rather than with early *Homo* (SK 847). *P. robustus* and SK 847 are most differentiated (at p < 0.01) along the largest component variance (PC1) in one PCA (Fig. S7), or along the second largest one (PC2) in the other PCA (Fig. 2c). Therefore, both landmark data and morphometric features (angles and indices) are taxonomically meaningful. The MDS (Fig. 2d) (with a stress value of 0.0068 which indicates a very good representation quality) is consistent with the PCA results and shows a very good fit of the specimens in their groups (“Methods”). As shown by the comparison between the minimum and maximum values along PC2 in the shape space (Fig. 2b), DNH 34 and DNH 60 share significant features with *P. robustus*, when compared to early *Homo* (SK 847) (Fig. 2c). In early *Homo*, the posterior SCC (PSC) is posteriorly expanded (Fig. 2b). The horizontal SCC (HSC) is reduced in relative diameter and its posterior part is superiorly displaced (Fig. 2b). Finally, the lateral extremity of the anterior SCC (ASC, near the center of the ampulla, landmark 5) is inferiorly displaced (Fig. 2b). Among the 12 indices and 10 angles of the BL (“Methods”, Table S3), the posterior semi-circular canal index 1 (PSCI1 that divides the arc length of the PSC situated below and above the HSC) (“Methods”) is the measurement that most differentiates *P. robustus* from SK 847 (i.e., with the most significant correlation with PC1, r = 0.88, p < 0.001, Fig. S7).

### Variation of the SCCs among extant and fossil hominins

**Figure 3:**
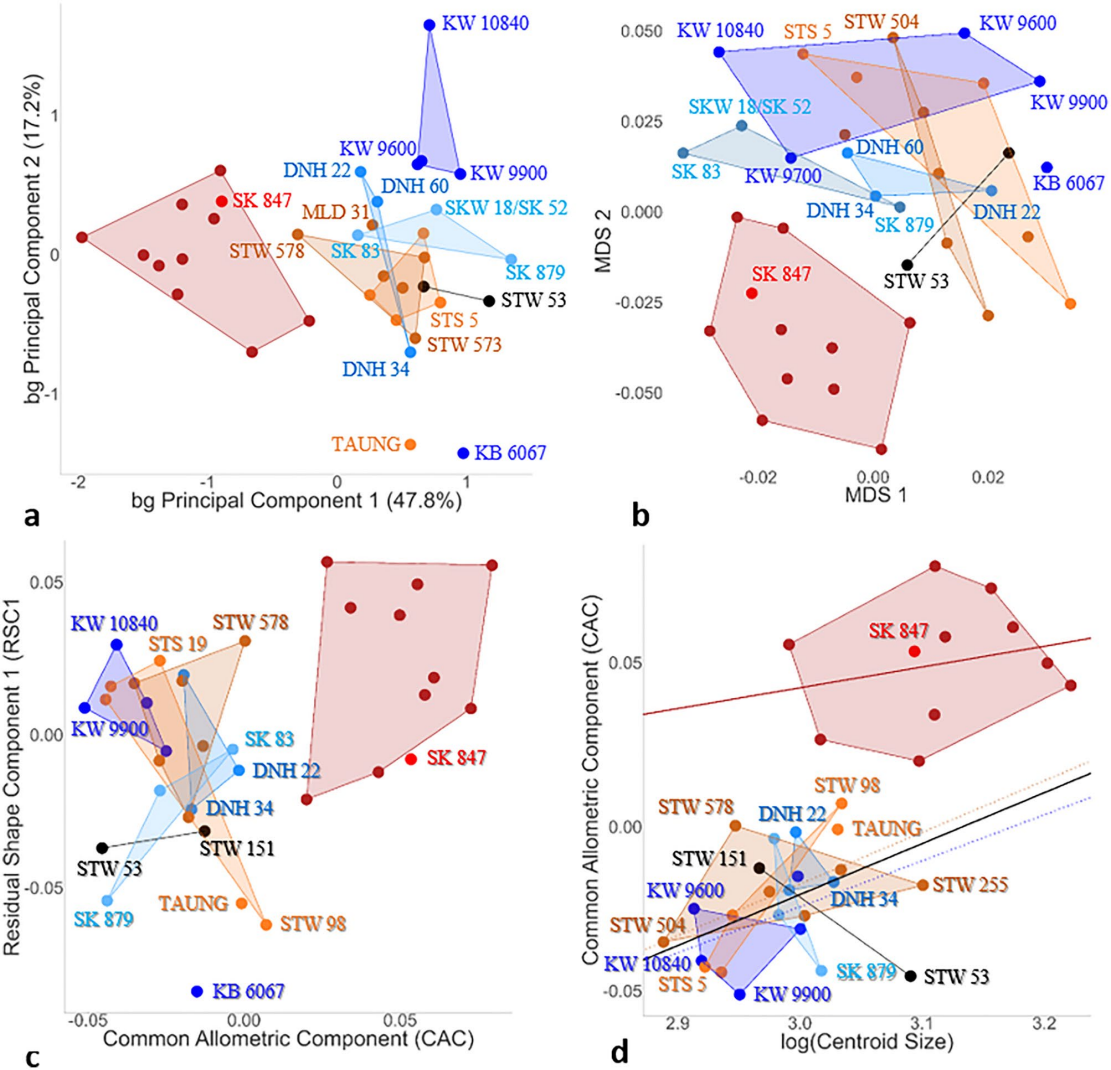
Assessments of semi-circular canal (SCC) variation among fossil and extant hominins using Procrustes and Mahalanobis distances after a Generalized Procrustes Analysis (GPA). (**a,b**) Morphospace obtained from either a between-group principal component analysis (bg PCA) after a GPA showing the bgPC1 versus bgPC2 when 7 semilandmarks per SCC are considered (**a**) or a multidimensional scaling (MDS) from computational anatomy showing MDS 1 versus MDS 2 (**b**) (“Methods”). (**c**) CAC versus RSC1 scores. (**d**) Log centroid size versus CAC (also illustrated by two separate linear regression lines; one for australopiths—in black—and one for *Homo*—in red; p < 0.01). *P. robustus* from the Kromdraai (n = 5, dark blue), Drimolen (n = 3, light blue) and Swartkrans (n = 2, light blue) sites, *A. africanus* (brown) from the Sterkfontein (n = 9), Makapansgat (MLD 31) and Taung (holotype) sites, early *Homo* from Swartkrans (SK 847, red), and modern humans (burgundy color) (n = 10 with equal numbers of females and males). Two fossil specimens (StW 151 and Stw 53, black) here considered as “indeterminate” are projected onto the biplots.

We use new fossil evidence from the sites of Drimolen (including DNH 34 and DNH 60) and Kromdraai to expand the knowledge of the variation of the SCCs within *P. robustus* and to compare it with *A. africanus* (Fig. 3). We consider the *A. africanus* (n = 11) and *P. robustus* (n = 11) specimens on the basis of their site and stratigraphic provenience (“Methods”). We also consider early *Homo* (SK 847) and one sample of modern *H. sapiens* (n = 10) (again, noting their similar SCCs^3^), and the StW 53 and StW 151 “indeterminate” specimens. We compute Procrustes and Mahalanobis distances after GPA^43^, and we illustrate them by a between-group PCA (bgPCA) (Fig. 3a) and a Canonical Variate Analysis (CVA)^44^ (as a type of discriminant analysis, Fig. S8), respectively (“Methods”). We also compute the MDS from the results of computational anatomy (Fig. 3b, stress value of 0.069) (“Methods”). StW 53 and StW 151 are projected onto each of the three biplots (Figs. 3a,b, Fig. S8). Because spurious patterns of differences between groups may arise when a bgPCA^45^ or a CVA^44^ is applied on small sample sizes relative to the number of variables, we assess this problem by varying the number of semilandmarks used to represent each SCC (1, 7, 18 or 48) (“Methods”, Fig. S9). Because the results do not change when using more semilandmarks (Fig. S9), we consider that 7 semilandmarks appropriately sample each SCC (Fig. 3a). The first two bgPCs (Fig. 3a) and the MDS (Fig. 3b) separate *H. sapiens* and early *Homo* on the one hand, and *P. robustus* specimens from Kromdraai Unit P on the other. All the other southern African australopiths occupy an intermediate position, with some overlap between *A. africanus* and *P. robustus* from Drimolen/Swartkrans (Fig. 3a) or from Kromdraai Unit P/Drimolen (Fig. 3b). We assess the morphological differences illustrated in Fig. 3a,b by using two methods. First, a Monte-Carlo test (10,000 permutations) (“Methods”) reveals that the differences reported in Fig. 3a reach statistical significance (p < 0.001) and that 46.3% of the total inertia comes from these differences (Fig. S10). Second, the fossil hominins illustrated in Fig. 3b fit very well in their respective groups, consistently with their site and stratigraphic provenience (Table 1), as indicated by the goodness of fit (0.069). The CVA also illustrates a separation between the three *P. robustus* samples (Fig. S8). Permutation tests (“Methods”) indicate that 100% of *P. robustus* specimens from Kromdraai Unit P and Swartkrans are correctly classified in their respective groups (Table S4). Two out of three *P. robustus* specimens from Drimolen are correctly classified (one is clustered with *Australopithecus*). The CVA results (Fig. S8) are therefore consistent with those of the bgPCA (Fig. 3a) and the MDS (Fig. 3b).

**Figure 4:**
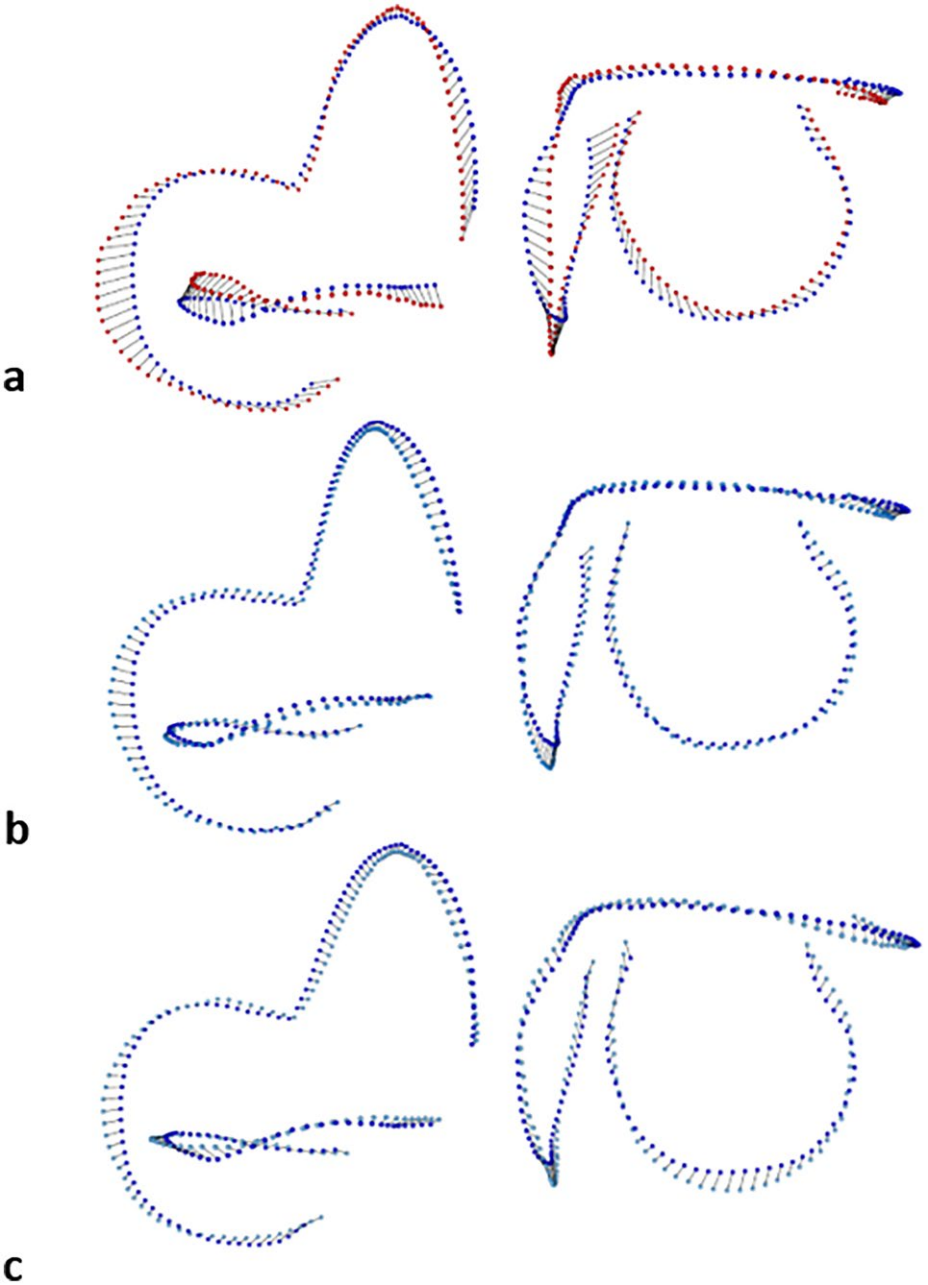
Shape differences between the SCC landmarks-semilandmarks configurations of *P. robustus* and early *Homo*. Differences shown with vector displacements, between the mean configurations of, on the one hand, *P. robustus* from Kromdraai Unit P taken as the reference (in dark blue) shown in posterior (left column) and lateral (right column) views with exactly the same orientations, and early *Homo* (in red) (**a**, top row), *P. robustus* from Drimolen (**b**, middle row) and Swartkrans (**c**, bottom row), on the other. To facilitate the comparisons, we represent each mean configuration with 48 semilandmarks per SCC (“Methods”) (Fig. S1).

In the bgPCA (Fig. 3a), the MDS (Fig. 3b) and the CVA (Fig. S8), StW 53 and StW 151 fall closer to *A. africanus* than to early *Homo* (Fig. 3). When compared to early *Homo* (Fig. 4a–c), *P. robustus* from Drimolen (Fig. 4b) and Swartkrans (Fig. 4c), the SCC mean shape sampled from Kromdraai Unit P is distinctive mainly because of its proportionally less developed PSC. Consistently with the results presented in Fig. 2b, the mean configuration from Kromdraai Unit P is typical of *P. robustus* when compared to SK 847.

### The SCCs in hominins versus African apes

We subsequently add a sample of African apes (Table S1) to investigate whether their SCC morphology can be used as a proxy for the ancestral hominin anatomy. It is indeed often considered that the australopiths and the African apes share the same SCC morphology that represents a condition similar to their common ancestor^13^. In order to investigate further the purported ancestral status of the African ape SCC, we project our fossil sample onto the bgPC1/bgPC2 morphospace computed by using modern African apes and humans only. Most australopiths fall within the lower end of the common chimpanzee SCC variation along bgPC1 (Fig. S11). However, some *A. africanus* and *P. robustus* SCCs evince a morphology not found in our samples of African apes and modern humans (Fig. S11). The MDS does not discriminate better these groups (Fig. S12).

### The evolutionary allometry of the SCCs

**Figure 5:**
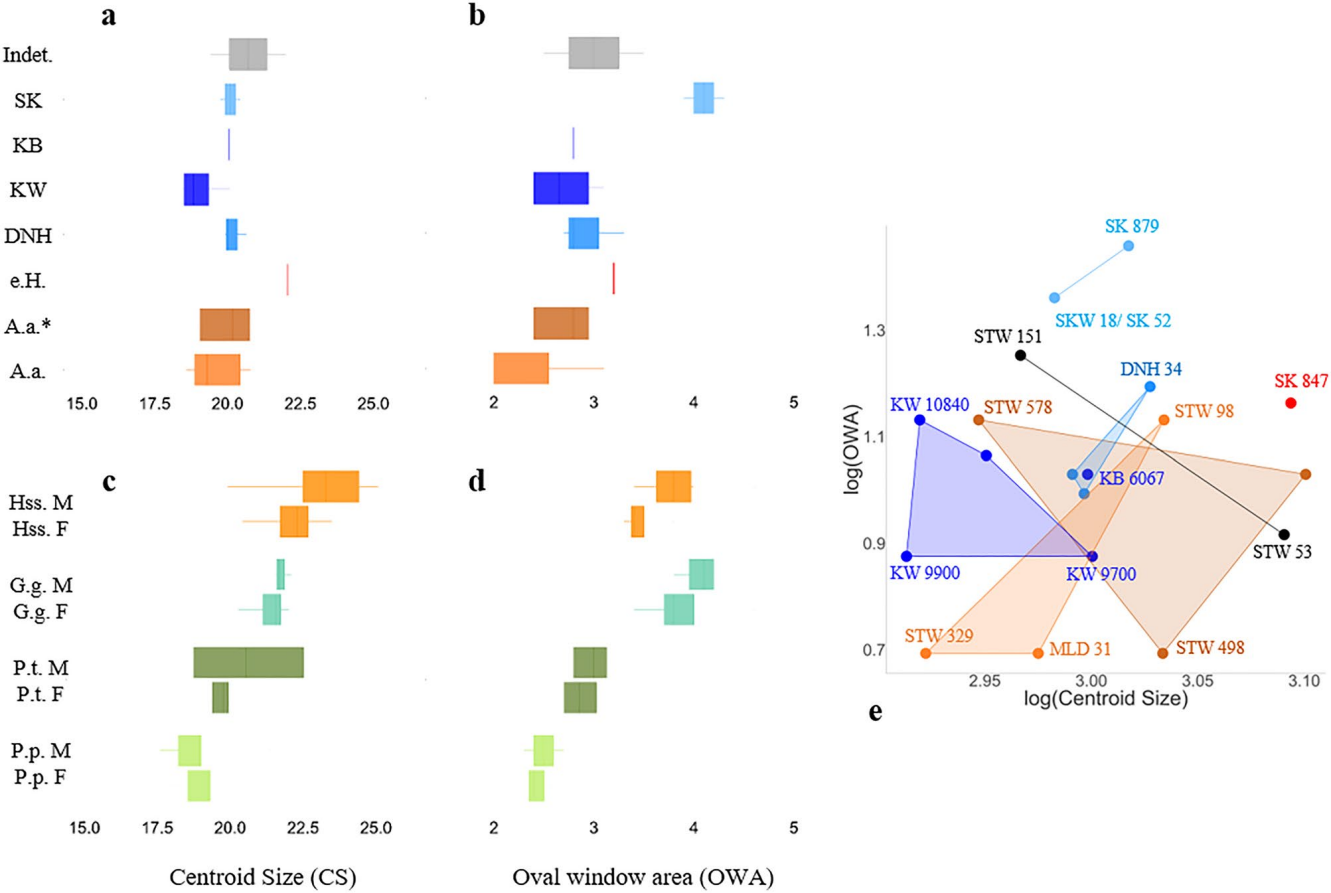
Oval window area (OWA) and centroid size (CS) of the SCC among southern African hominin and modern species. **(a–d)** Box plots showing the OWA (**b,d**) and CS values (**a,c**), and biplot of log centroid size (CS) versus OWA (**e**) in fossil specimens (**a,b**)—*P. robustus* from Kromdraai Unit P (KW, dark blue), Unit Q-R (KB, dark blue), Swartkrans (SK, light blue) and Drimolen (DN, light blue), early *Homo* (e.H., red), *A. africanus* (A.a. black, * is for specimens that have been referred to a second species with purported robust australopith affinities^7^), and two fossil considered as “indeterminate” (StW 53 and StW 151, grey)—and in four modern species (c,d): modern humans (Hss, orange), bonobos (P.p., light green), common chimpanzees (P.t., dark green), gorillas (G.g, cyan). *F* female, *M* male.

Allometry has been an essential concept for evolutionary biology because variation in size among taxa is an important determinant for their morphological variation^21^. Here we examine the evolutionary allometry of the SCCs, i.e. the shape variation that is associated with differences in size between species. We therefore separate the SCC shape differences caused by allometry (CAC) from those that are not (RSC1)^11^ (Fig. 3c). The SCC shape in *P. robustus* from Kromdraai Unit P is distinctive either because of allometric scaling (along CAC) when compared to Drimolen, or independently of size (along RSC1) when compared to Swartkrans and KB 6067 (Fig. 3c). In the hominin sample, there is a significant linear correlation between CAC and SCC centroid size (the measure of SCC size used to scale its configuration of landmarks) (r^2^ = 0.117, p < 0.001) (Fig. 3d), a relationship that even improves when the specimens are grouped according to their species and site/stratigraphic provenience (r^2^ = 0.55, p < 0.001) to create an additional covariate (Wilcoxon tests, p < 0.05). The CAC versus RSC1, and SCC centroid size versus CAC relationships observed among fossil hominins are confirmed when the four modern species are added to the model (Fig. S13). The lower CAC values observed in *P. robustus* from Kromdraai Unit P correspond to its smaller SCC centroid size (mean and individual values) (Figs. 3d and 5a). However, none of the differences in SCC centroid size between the fossil samples are significant (Fig. 5a, Table S5). On the contrary, most of the differences in SCC centroid size between modern species are significant, with no sexual dimorphism (Fig. 5a, Table S5). Therefore, allometry is important in differentiating SCC shape in *P. robustus* specimens from different site/stratigraphic provenience, but size differences are caused neither by sexual dimorphism, nor by ontogeny. For instance, within *A. africanus*, its holotype (Taung child) shows higher CAC and SCC centroid size values than StW 504 (Fig. 3d), a presumably large male^16^. In *P. robustus* from Kromdraai Unit P, the KW 9900 adult also shows lower CAC and SCC centroid size values than the KW 9700 child (Fig. 3d).

### The oval window

The oval window is located between the middle ear and the cochlear part of the inner ear. Measurements of the oval window area (OWA) may be correlated with body size within primate species^46,47^. We therefore give particular attention to repeatable measurements of the OWA (“Methods”) in order to prevent spurious results, as emphasized previously^46^. *P. robustus* specimens from Kromdraai/Drimolen show a significantly lower OWA than those from Swartkrans (p < 0.05, Wilcoxon). An example of the difference in OWA between specimens from Kromdraai/Drimolen (DNH 22) and Swarkrans (SK 879, with its 59% larger OWA) is illustrated in Figure S14. The OWA mean value from Kromdraai/Drimolen (n = 8) falls 3.5 standard deviations below the smaller OWA value from Swartkrans (Fig. 5b,d,e). Ontogeny does not influence differences in OWA among australopiths, larger values being often measured in juveniles (e.g., KW 10840 and StW 98) than in adults, as already noticed in modern humans and African apes^46,47^. There are significant differences in OWA between, on the one hand larger-bodied modern humans and *G. gorilla* (with no significant difference between them), and *P. troglodytes* or *P. paniscus* on the other (Fig. 5b), with no sexual dimorphism within any of these four modern species (Table S5). Among australopiths, there is no correlation between the oval window area and SCC centroid size (p = 0.62). The SCC centroid size versus the oval window area biplot shows a separation between the three *P. robustus* samples (Table 1), the Kromdraai Unit P specimens falling in the lower left quadrant with lower values (Fig. 5d).

## Discussion

The main goal of this study is to test whether and how distinct *P. robustus* groups can be distinguished consistently with their site and stratigraphic provenience. We find a site-related diversity in *P. robustus* SCC shape that is partly attributable to differences in size (allometry) (Fig. 3c). Some SCC shape differences between *P. robustus* from Drimolen and Kromdraai Unit P (Fig. 3c) are caused by a smaller SCC centroid size in the latter sample (Fig. 5a). The SCC shape uniqueness of the Kromdraai Unit P *P. robustus* (Fig. 4) is consistent with the well-known allometric trend in which larger-bodied primates possess a proportionally more developed posterior SCC^35^. However, the smaller SCC centroid size in specimens from Kromdraai Unit P is not necessarily associated with differences in body size between the three *P. robustus* samples for several reasons. First, SCC shape is also influenced by locomotor agility in primates in general^48^. Second, the differences between the Kromdraai Unit P and Drimolen *P. robustus* samples might reflect sexual dimorphism because SCC size was found to be significantly larger in *G. gorilla* males than in females^37^. Turning to variation in SCC centroid size among the four extant species investigated in our study, we nevertheless find no sexual dimorphism in *Pan*, modern humans, and even *G. gorilla*. It is also very unlikely that the *P. robustus* specimens with small and large SCC centroid size represent females from Kromdraai Unit P and males from Drimolen, respectively. Indeed, under the assumption of a balanced sex ratio, the probability that the four *P. robustus* specimens from Kromdraai Unit P represent only females is low (0.5^4^ = 0.0625). Given that *P. robustus* females are also represented at Drimolen^8^, it therefore seems reasonable to consider a random sampling of sex ratio in both this latter site and Kromdraai. Because the distinctive SCC shape measured in *P. robustus* from Kromdraai Unit P is also size-independent (i.e., not entirely caused by allometry, as indicated by MDS—Fig. 3b—and the CAC versus RSC1 biplot, Fig. 3c), the site-related diversity in *P. robustus* SCC likely has another underlying cause than sexual dimorphism in body size.

As emphasized earlier, differences in size between *P. robustus* craniodental specimens are most often interpreted as sexual dimorphism in body size^22^. Therefore, another matter of importance for a better understanding of *P. robustus* paleodiversity is the study of differences in the size of traits other than those of the facial skeleton and teeth, by controlling for geographic variation and sex. Our results underline the importance of the oval window area in the analysis of *P. robustus* paleodiversity. The comparison of the OWA values, consistently with our results on SCC centroid size and shape, indicates a site-related diversity in *P. robustus.* The smaller SCC centroid size in the Kromdraai Unit P specimens is associated with a significantly smaller OWA in the Kromdraai/Drimolen *P. robustus* sample, when compared to the one from Swartkrans (Fig. 5, Table S5). Given that sexual dimorphism and geographic variation are not involved (see above), the smaller OWA shared between *A. africanus* and the Kromdraai/Drimolen *P. robustus* samples (Fig. 5) might represent a more primitive (plesiomorphic) condition for the *P. robustus* lineage. The smaller OWA and SCC centroid size measured in the *P. robustus* samples from Kromdraai Unit P and Drimolen (Fig. 5) is also consistent with the overall small size of both the maxillary dentition and the temporal bone of KW 9900 by *P. robustus* standards. Indeed, KW 9900 defines the low end of variation in *P. robustus* in two MD diameters of its teeth (P4 and M2) (Table S2). The dimensions of the KW 9900 temporal bone are smaller than in any other *P. robustus* specimen. When compared to the subadult and type specimen of *P. robustus* from Kromdraai, the geologically older KW 9900 specimen also appears particularly small in its dental and temporal bone dimensions (Text S1). The differences between TM 1517 and its conspecifics from Swartkrans (e.g., SK 46 and SK 83), on the one hand, and the smaller, presumably *P. robustus* female (DNH 7) from Drimolen, on the other hand, have been interpreted as consistent with sexual dimorphism within *P. robustus*^22^. If these differences in size are interpreted exclusively in terms of sexual dimorphism, this would imply that KW 9900 simply represents a small female of *P. robustus*. However, again, the three other *P. robustus* specimens from Kromdraai Unit P investigated in this study (KW 9600, KW 9700 and KW 10840) also appear particularly small (Figs. 3d, 5a,e) and the probability that they all represent females is very low (see above).

As for the morphological traits visible on the outer aspect of the temporal bone and considered to describe an ancestral-descendant sequence among *P. robustus*^8^, the KW 9900 small specimen from Kromdraai Unit P evinces features that are unlike those of any small or large specimens from Swartkrans and Drimolen. For instance, the KW 9900 anteriorly positioned mastoid tip is unlike the two other adult *P. robustus* fossils from Swartkrans (SKW 18/SK 52) and Drimolen (DNH 7) in which this trait can be assessed (Text S1), and more similar to the plesiomorphic condition observed in *A. afarensis* and *A. africanus*^8,41^. Moreover, the absence of a lateral extension, widening and flaring of the lateral portion of the KW 9900 tympanic bone (Figs S3-4) is shared with the DNH 7 skull and was interpreted as plesiomorphic^8^ (Fig. 5). The observations presented here will need to be supplemented by new approaches applied to the most complete specimens of *P. robustus* (e.g., DNH 7) including its type specimen from Kromdraai (TM 1517). For the moment, in the absence of high resolution (i.e., with pixel size lower than 50 microns) and sufficiently contrasted micro-CT data, important inner features of the TM 1517 temporal bone (including OWA and cochlear shape) cannot be accurately measured.

In light of these findings, it is also interesting to discuss further the status of the KB 6067 specimen from Kromdraai Unit Q-R^3^. When compared to other *P. robustus* specimens available at this time, the smaller OWA in KB 6067 was interpreted as a possible primitive condition for this species, with similarities to *A. africanus*^3^. More recently, KB 6067 was confirmed to be *P. robustus* on the basis of its robust-like and size-independent cochlear shape^4^. The KB 6067 SCC shape is distinctive when compared to its conspecifics from Kromdraai Unit P, and to other *P. robustus* specimens from Drimolen and Swartkrans (Fig. 3). This difference is size-independent (i.e., caused mainly by RSC1) (Fig. 3c). Interestingly, and as shown by the degree of opening of its subarcuate fossa (20%), KB 6067 is developmentally much younger than any other *P. robustus* child in our sample (KW 9600:7.2%, KW 9700:6.4%) and closer to the StW 98 (17.8%) very young *A. africanus* infant^3,4^. Therefore, the size-independent SCC shape in KB 6067 (its RSC1 value shown in Fig. 3c is closer to StW 98 than to any other australopith) may be affected by some, yet unknown, prenatal or perinatal growth affecting its development. Further investigation is required to evaluate this hypothesis.

In the present study, the SCCs of StW 53 and StW 151 show closer similarities with *A. africanus* than with SK 847, the only undisputed early *Homo* specimen in our sample. StW 53 has been attributed to early *Homo*^49^, but previous analyses revealed unique features of its bony labyrinth^13^, its *Australopithecus*-like cochlear shape^4^ and the late fusion between its maxillary and incisive bones^50^. When other cranial features have been considered sufficiently diagnostic to retain StW 53 within early *Homo*^49^, they have been visually assessed and classified into vague categories (e.g., ‘narrow’ versus ‘moderate’, or ‘short’ versus ‘short/moderate’). We therefore argue that more detailed morphological analyses of StW 53 cranial remains are needed to test statistically the taxonomic status of this specimen. From the results of the present study, we conservatively assume that it does not represent early *Homo* because its BL is too plesiomorphic when compared to SK 847.

The cochlea of early *Homo* resembles that of *A. africanus* and, in both of these two taxa, it is intermediate between *P. robustus* on the one hand, and modern humans, chimpanzees and gorillas on the other^4^. Importantly, SK 847 displays a mosaic of features that combines modern human-like SCCs^3,13^ and an *Australopithecus*-like cochlear shape^4^. The cochlear shape yields a different evolutionary signal than the SCC among southern African early hominins. The uniquely derived and invariant cochlea of *P. robustus* reveals a strong selection for its morphology early in the evolutionary history of this species^4^. This evidence may be seen as incongruent with the SCC pattern of diversity in *P. robustus* and also reveals a modular (or mosaic) evolution of the bony labyrinth in southern African early hominins.

Radiometric dates and biochronological data from the uncorrelated sedimentary sequences of Kromdraai, Drimolen and Swartkrans are to be consistent in order to allow further interpretations of the *P. robustus* site-related paleodiversity described in this study. However, as yet, none of the age estimates from these sites can be taken into account with certainty due to limited multi-proxy dating approaches, analytical error ranges associated with absolute dating methods, and the paucity of biochronological data from Drimolen^8^. When compared to other robust australopiths from southern Africa, *P. robustus* from Kromdraai Unit P is nevertheless distinctive because of its smaller and more plesiomorphic adult temporal bone, its smaller oval window area and semi-circular canals, and its proportionally less developed posterior SCC, independently of age and sex. Uncertainties surrounding the dating of *P. robustus* from Kromdraai Unit P would not disqualify this sample from being close to the root of this species lineage, as shown by its more plesiomorphic features. We therefore consider that the *P. robustus* site-related differences reported in this study may result from evolutionary changes within this lineage.

## Methods

Details on the provenience and the sex (when available) of the specimens in our samples, and the micro-CT systems used to scan them, are provided in Table S1. Among the 25 southern African early hominins, one juvenile (DNH 34) and one adult (DNH 60) specimen from Drimolen were previously unpublished, and one adult (KW 9900) and three juvenile (KW 9600, KW 9700, KW 10840) *P. robustus* specimens from Kromdraai Unit P were described only for their cochlear shape^4^. The DNH 34 juvenile status is indicated by the degree of the opening of its subarcuate fossa (5.6%) that is equivalent to that measured in the KW 9600 and KW 10840 specimens^4^. Here, we describe these six new specimens from Drimolen and Komdraai Unit P by focusing on their taxonomically diagnostic features. Among the other fossil hominins in our sample, all have already been published. However, the BL of some of these published specimens is newly reconstructed and investigated in the present study. This is the case for one *P. robustus* (SK 83) and two *A. africanus* (Taung child, MLD 31) specimens. As demonstrated both previously^4^ and in the present study, there are no differences in BL size and shape between adult and juvenile specimens among hominid species, and in other placental mammals^39,51^. We therefore combine juvenile and adult specimens in our samples.

The majority of the specimens in our samples are represented by their right BL. In cases of damage of the right side, the left BL is reconstructed after mirroring, with no loss of size and shape information during this process. Mesh surfaces of each BL were obtained in Avizo Standard 8.1.1 (https://www.thermofisher.com) from micro-CT data resliced in a plane that best fitted the horizontal SCC. The measurement protocol of the SCC began with the computation of the centerlines of the SCCs from the surface models with the ‘Skeletonization’ pack and the ‘Autoskeleton” module. A set of five landmarks was digitized on each centerline skeleton (Fig. 1a) and the placement error was estimated (see below). Landmarks 1 and 2 (Ld1 and Ld2, respectively) are located on the horizontal SCC (HSC) and correspond to the intersections between the plane that best fit the anterior SCC (ASC) and, respectively, the lateral and medial ends of the HSC’s centerline. Landmark 3 (Ld3) is located on the center of the ampulla of the posterior SCC (PSC). Landmark 4 (Ld4) is the bifurcation point of the common crus and landmark 5 (Ld5) is the center of the ampulla of the ASC. We also placed one landmark at the apex of the cochlear curve (Landmark 6, Ld6).

We then assess the size and shape of each BL either by a geometric morphometric method (GMM) (see below), by a deformation-based method from computational anatomy (see below), or by using 35 variables that include one area, six arc lengths, six linear distances (line segments), 12 indices and 10 angles described in Text S2 and detailed in Table S3. Among these 35 variables, five have been defined in previous studies^3,13^. All these 35 variables but one (OWA) are measured directly on each centerline skeleton by using the best-fitting planes of the HSC (HSCP) and ASC (ASCP), and the surface mesh. All these 35 measurements are described in Text S2 and provided in Supplementary Material (‘Data S1.xls’ in ‘Datasets’).

For GMM, we used the R packages Morpho 4.0.5 (see https://cran.r-project. Org/web/packages/Morpho/index.html) and Geomorph version 4.0.0 (see https://cran.r-project.org/package=geomorph). We considered four varying sets of semilandmarks (1, 7, 18 or 48) to represent each SCC. Therefore, we conducted four different analyses by using a total of either 8, 26, 59 or 149 (semi)-landmarks (Fig. S9). These (semi)-landmark data are provided in Supplementary Material (‘Data S2–7.csv’ in ‘Datasets’).

We first performed a Generalized Procrustes analysis (GPA) (with scaling). Following superimposition, we summarized variation in shape space by using a Principal Components Analysis (PCA) and a between-group PCA (bgPCA). We use a permutation test (or Monte-Carlo test, or randomization test) in the R ade4 package (see https://cran.r-project.org/web/packages/ade4/index.html) in order to assess the statistical significance of the bgPCA. The statistical significance is evaluated with the ‘randtest.between’ function by simulating 999 permutations.

To complete our analysis, we additionally analyze our sample using a deformation-based method from computational anatomy. Instead of relying on sparse corresponding features such as GMM, this method estimates and analyzes dense deformations (diffeomorphisms) between non-homologous curve and shapes. For pairwise alignment of the SCCs, we employ deformetrica 4.0 (www.deformetrica.org)^52^. The alignment is driven by the metric of currents, which enables a comparison of non-homologous curves. The difference between the SCCs of two specimens is then modeled as the amount of diffeomorphic (smooth and invertible) deformation needed to align them. A more detailed description of the method and application to the shape analysis of fossil hominins can be found in reference 11. Each diffeomorphism is modeled as a vector field that describes the displacements of a regular grid of control points, deforming the underlying space. The displacements of each control point represent the estimated amount of deformation between two curves. For our analysis, we compute a symmetric distance matrix from the control point displacements by calculating each pairwise distance as the average L2-norm of the displacement vectors. We performed a non-metric Multidimensional Scaling (MDS)^53^ with an embedding dimension of two, in order to embed the high-dimensional data in a low-dimensional embedding space using the scikit-learn library (version 0.24.2) in Python 3.8.10.

Since we investigate multiple species across a wide range of sizes, the first modes of variation on the PCA and bgPCA, likely represent a combination of size-correlated shape differences and shape differences among taxa unrelated to size^10,44^. We therefore investigate the relationships between SCC shape and size changes (allometry). The common allometric component (CAC) represents the pooled within-group direction of covariation (after removing inter-group variation) between the shape variables on the log centroid size^10,44^. The RSC scores describe the residuals of the pooled regression analysis, i.e., the non-allometric component. The first principal component of this analysis is referred to as residual shape component 1 (RSC1)^10^. We explore variation in the common allometric component (CAC) and residual shape components (RSCs)^10^. Finally, we employ the first few PC axes in the calculation of Canonical variate analysis (CVA) that maximizes the between-group variance relative to the within-group variance in order to differentiate a priori defined groups. In all the statistical analyses, because the StW 53 and StW 151 specimens are considered as indeterminate, we project them onto the (bg)PC1/(bg)PC2 representations in order to identify their closest neighbours. This is also the case for the DNH 34 and DNH 60 specimens when we first investigate their taxonomic (i.e., before their attribution to *P. robustus*) (Fig. 2). We differentiate nine a priori defined groups of fossils (Table 1, Table S1) in the statistical analyses. We define four *P. robustus* groups: (i) one from Kromdraai Unit Unit P (KW 9600, KW 9700, KW 9900, KW 10840), (ii) one specimen from Kromdraai Unit Q-R (KB 6067), (iii) three from Drimolen (DNH 22, DNH 34, DNH 60) and (iv) three from Swartkrans (SK 83, SK 879, SKW 18/SK52) (Table 1). We also define four *A. africanus* groups: (i) the holotype from Taung, (ii) one specimen from Makapansgat (MLD 31, (ii) two samples from Sterkfontein in order to distinguish the specimens attributed to this species on a consensual basis (Sts 5, Sts 19, StW 98, StW 329) from those (StW 252/255/259, StW 498, StW 504/505, StW 573 and StW 578) that have been referred by one researcher^29^ to a second species with purported robust australopith affinities (Table 1).

### Ethical approval

All the steps of the present study were performed in accordance with relevant guidelines and regulations. No data used in this study involved experimentation, risk or constraint added by the research. Only museum specimens were included in this study and we obtained permissions to access them.

## Supplementary Information

Supplementary Information 1.

Supplementary Information 2.

Supplementary Information 3.

Supplementary Information 4.

## Data Availability

The datasets generated and/or analyzed during the current study and the codes used in the analysis are available as Supplementary Data. The Data_S1-7 files are available in one compressed file (‘Datasets.zip’). These data were processed by using the R Code available in the ‘R code’ file. The data preparation script and code used for computational anatomy (shape matching and analysis) are available in another compressed file (‘Python code’). Original new fossils from Kromdraai and Drimolen, and other fossils with the catalogue prefix StW, are curated at the Evolutionary Studies Institute, University of the Witwatersrand in Johannesburg, South Africa, and researchers may apply for access through the University Fossil Access Advisory Committee by contacting the University Curator for Fossil and Rock Collections: Bernhard Zipfel, PhD, University Curator of Fossil and Rock Collections, Evolutionary Studies Institute, University of the Witwatersrand, Johannesburg (e-mail: Bernhard.Zipfel@wits.ac.za; Phone: + 27-11 717-6683). For access to the original comparative fossils with the catalogue prefixes TM, SK and Sts, these are curated at the Ditsong Museum of Natural History in Pretoria, South Africa, and researchers may apply for access to Fossil Access Committee by contacting: Mirriam Tawane, PhD, the Curator of Plio-Pleistocene Palaeotology, Ditsong Museums of South Africa (e-mail: tawane@ditsong.org.za; Phone: + 27-12 492 5744).
