# Supplementary material for: New fossils from Kromdraai and Drimolen, South Africa, and their distinctiveness among Paranthropus robustus
Source: Sci Rep. 2022 Aug 17;12:13956. doi: 10.1038/s41598-022-18223-7 (PMC9385619; doi:10.1038/s41598-022-18223-7)
Supplement: Supplementary file 4 — Supplementary Information 4. [file 41598_2022_18223_MOESM4_ESM.docx]

**Supplementary Information for**

**New fossils from Kromdraai, South Africa, and their distinctiveness among *Paranthropus robustus***

J. Braga^1,2*^, G. Chinamatira^2^, B. Zipfel^2^, V.A. Zimmer^3^

1. Centre for Anthropobiology & Genomics of Toulouse, CNRS UMR 5288, Université de Toulouse, Université Paul Sabatier, 37 allées Jules Guesde, Toulouse, France.

2. Evolutionary Studies Institute, University of the Witwatersrand, PO WITS, Johannesburg, 2050, South Africa.

3. Faculty of Informatics, Technical University of Munich, Munich, Germany.

* Corresponding author: [jose.braga@univ-tlse3.fr](mailto:jose.braga@univ-tlse3.fr)

**Contents of this file (31 pages)**

Texts S1-2

Figures S1-S14

Tables S1-S5

Supplementary References

**Text S1**

**Anatomical descriptions**

We provide anatomical descriptions and illustrations of the temporal bones of one juvenile (DNH 34) and one adult (DNH 60) unpublished specimens from Drimolen Main Quarry, one adult (KW 9900) and one juvenile (KW 10840) *P. robustus* specimens from Kromdraai Unit P. The two later specimens have been partly described in a study of their cochlear shape^4^. Here, we focus only on the taxonomically most important morphological features.

DNH 34 (Fig. S1). The DNH 34 specimen was attributed to early *Homo* without explanation^40^. One fragment preserves most of the right petrous (Fig. S5). The tympanic is preserved from its anterior to its posterior walls. The DNH 34 juvenile status is measured on the isolated petrous part of its right temporal bone. The degree of the opening of the DNH 34 subarcuate fossa (5.6%) is similar that of the KW 10840 infant^4^. Another fragment is the basilar part of the occipital bone (pars basilaris) with its completely unfused synchondrosis intraoccipitalis anterior (on both sides) that confirms the juvenile status of DNH 34. The pars basilaris is short (LB:11.0 mm) and wide (WB:14.3 mm) with an index (WB/LB x 100) of 130 (for definitions of LB and WB, see Fazekas and Kosa, 1978). In comparison, the WB/LB index is lower in the juvenile L 338 y-6 *P. boisei* specimen (111) from the Shungura Formation (Member E) in the Omo succession (Ethiopia), and in the juvenile STW 187a *A. africanus* specimen (106) from Member 4 at Sterkfontein.

As in KW 10840, the external auditory meatus of DNH 34 is sub-elliptical in shape (major axis is 11.4 mm long, and its minor short axis is 8.2 mm), with its long axis oriented obliquely, with its inferior end set anteriorly. The anterior face of the anterior wall of the tympanic bone is also separated from the unpreserved postglenoid process of the mandibular fossa by a distinct groove. As in KW 10840, the slightly concave inferior face of the tympanic bone of DNH 34 is rectangular in shape, and appears longer antero-posteriorly 13.0 mm. The thin inferolateral edge of the tympanic bone is also (as in KW 10840) much recessed medially and leaves a distinct oval hollow laterally, a feature also reflecting its young age (see above).

The absence of an ossified styloid process fused with the exocranial surface of the petrous bone is likely the consequence of the young age of DNH 34. This reflects the absence of ossification of the cartilaginous band persisting between the tympanohyal part and the larger stylohyal part (forming the major part of the future osseous styloid process) (Braga, 1993).

DNH 60 (Fig. S1). The DNH 60 specimen was also attributed to *P. robustus* without explanation^40^. It consists only in the isolated petrous part of the right temporal bone that show no taxonomically diagnostic features.

KW 9900 (Fig. S2-5). The KW 9900 specimen is fully adult. It was attributed to *P. robustus* on the basis of its cochlear shape and the overall proportions of its semi-circular canals (SCC)^4^. KW 9900 preserves two cranial fragments associated with the isolated albeit associated complete maxillary dentition. The larger cranial fragment preserves most of the three components (petrous, tympanic and squamous) of the right temporal bone articulated with a short fragment of the right parietal. The smaller cranial fragment preserves the left petrous part of the temporal bone and the left mastoid process.

The parietal fragment, in an area closest to the region of the parietal eminence, has a thickness of 4.6 - 5.5 mm. The bone is thinner (4.0 mm) immediately superior to the squamosal suture.

The temporal bone is preserved from the squamosal suture to the mastoid region, the petrous part and the entire tympanic bone (Fig. S3-4).

The squamous portion is preserved intact superior to the external auditory meatus. The well- preserved and very thin margin of the squamosal suture largely overlaps the parietal. It extends from the damaged root of the zygomatic arch anteriorly, to the broad, flange-like supramastoid crest. Its margin is superiorly convex, and its highest point is located about 24 mm above the supramastoid crest and about 34 mm above the roof of the external auditory meatus.

The lateral aspect of the squamous portion and mastoid process is marked by a prominent supramastoid crest that forms a projecting flange separated from the squamous plate by a wide gutter (ca. 7 mm) and a marked supramastoid sulcus (ca. 2.5 mm) (Fig. S4). The supramastoid crest thickens anteriorly. Its inferior margin follows an anteroinferior course to blend into the posterior wall of the external auditory meatus. Its superior border becomes confluent anteriorly with the suprameatal crest and the damaged posterior root of the zygomatic process. The supramastoid crest is separated from the blunt mastoid crest below it by a shallow (ca. 3 mm) supramastoid sulcus that continues antero-inferiorly to form the tympanomastoid furrow that extends behind the tympanic plate. In the TM 1517 specimen, the supramastoid crest is less developed than in KW 9000, and is not separated vertically from the mastoid crest as in KW 9900. The inferior surface of the mastoid process forms a rugose and broad, very slightly convex face for the insertion of the sternocleidomastoid, splenius capitis and longissimus capitis muscles. Although the apex of the mastoid process (or mastoid tip) is missing, the preserved remnant projects below the external auditory meatus and, anteriorly, reaches a level just posterior to the level of porion (Fig. S4).

The asterion is not preserved. The minimum porion-asterion distance is 38 mm. The maximum porion-mastoid distance is 10 mm. Therefore, the “mastoid tip position index”^41^ in KW 9900 is lower than 30%, an estimation lower than that of the two values yet reported for *P. robustus* (32% and 38% in SKW 18/SK 52 and DNH 7, respectively^8^).

The well preserved postglenoid process project approximately 12.5 mm below the superior margin of the zygomatic root, a dimension that is notably smaller than in TM 1517 (17 mm) and any of the *P. robustus* specimens from Drimolen (DNH 7 16 mm) and Swartkrans (20-22 mm, Wood, 1991). This features in KW 9900 compares more favorably with *A. africanus* specimens (11-16 mm; Wood, 1991).

The external auditory meatus shows a nearly vertical anterior wall bounded superiorly and anteriorly by a deep supraglenoid gutter. The meatus is an ovoid opening (its nearly vertical major axis is 12.4 mm long, and its minor short axis is 9.2 mm), and its size (88.4 mm^2^, calculated from the area of an ellipse) is similar to the dimension reported in the presumably *P. robustus* female cranium from Drimolen (DNH 7), but notably smaller than in TM 1517 (125 mm^2^, pers.obs.) or any of the three *P. robustus* specimens from Swartkrans (> 125 mm2 in SK 46, SK 83 and SKW 2581, Wood, 1991).

The anterior wall of the tympanic plate is slightly concave mediolaterally. Its anterior face is separated laterally from the postglenoid process and medially from the posterior wall of the mandibular fossa by a distinct groove. Unlike most *P. robustus* specimens, in KW 9900, the relatively thin inferolateral edge of the tympanic bone (2.7 mm) is slightly recessed from the lateral margin of the mastoid process and the suprameatal crest, and therefore does not attain its maximum lateral extension at this level (Fig. S3-4). When seen in inferior view, the tympanic is rectangular in shape with nearly parallel anterior and posterior border (Fig. S3-4). This morphology contrasts with the typical “trumpet” shape of the tympanic bone described in *P. robustus*, including its holotype (wider and flared laterally, and narrowing toward the middle when seen in inferior view)^42^ to the exception of DNH 7^8^. The distance from the lateral margin of the tympanic bone to the carotid foramen (CC-LT, as defined by Dean and Wood, 1982) is 22.1 mm. This relatively short length of tympanic bone falls within the range of values in *A. africanus* and *A. afarensis*, and well below the ranges measured in the SK 47 and TM 1517 (25.0 and 35 mm, respectively) (Dean and Wood, 1982) and in the DNH 7 (30 mm)^8^ *P. robustus* specimens.

Unlike most *P. robustus* crania (e.g., SK 46, SK 48, SK 52, SK 848, TM 1517, DNH 7), the tympanic bone of KW 9900 does not contribute to the inferior part of the posterior wall of the external auditory meatus, which is formed entirely by the anterior face of the mastoid process. In this regard, it is similar to the condition described for A.L. 444-2^41^.

In KW 9900, the isolated crowns and roots of the maxillary dentition are preserved. Although they were found in anatomical position (Fig. S9), nothing of the maxilla is preserved, save for a few small pieces of bone that are wedged between the molar roots. The teeth are heavily worn; especially anteriorly, with the incisors and left canine crowns having been reduced in height nearly to the cervix (Fig. S5). The LP3 is also heavily worn, displaying a strong lingual concavity. The RC and RP3 are somewhat less heavily worn, but with the loss of all crown morphology. The L&R M1s are incomplete; the LM1 is the better preserved of the two. The L&R (especially L) M2s have deeply cavitated dentine wear exposures on the protocone, while the remainder of the crown enamel is worn flat but without any dentine exposure. The RM2 has a large antemortem enamel chip that was flaked from the lingual side of the dentine exposure. The L&R M3s show enamel wear across entire occlusal surface but without dentine exposure.

Owing to wear and damage, no meaningful details can be provided for the I1-M1, and very little can be said about the M2 or the M3 (Fig. S5). Nevertheless, the BL diameters can be recorded for almost all of the crowns, and the MD diameters can be reasonably estimated for the molars (Table S2).

The crowns of the maxillary second molars present a roughly square to rectangular (BL elongated) outline that is somewhat elongated BL. The four principal cusps are present. The protocone is the largest, followed by paracone and metacone, which are nearly equal in size. The hypocone is the smallest cusp. The crown is BL broader across the trigon than across the talon. The crowns of the maxillary third molars have a trapezoidal outline with rounded corners. The four principal cusps are present, but the hypocone is considerably reduced and takes the form of an irregular talon, where the distal marginal ridge (DMR) and cusp are not clearly separated. The protocone is considerably larger than the paracone and metacone, which are nearly of equivalent size to one another. The crista obliqua is thick and continuous. The DMR is incomplete on the left crown, where the talon basin, which takes the form of a BL-oriented furrow, is open at its buccal end. The buccal face is featureless and buccal groove is very shallow. The lingual surface of protocone evinces a small Carabelli pit.

KW 10840 (Fig. S6). The KW 10840 juvenile status is indicated by the degree of the opening of its subarcuate fossa^4^ that represents between 7.5% and 4.2% of the arc width of the anterior semicircular canal on the petrous part of its right and left temporal bone, respectively. This juvenile specimen was attributed to *P. robustus* on the basis of its cochlear shape and the overall proportions of its semi-circular canals (SCC)^4^.

The left temporal bone of KW 10840 is the better preserved and represents the only nearly complete temporal bone of a *P. robustus* child known (Fig. S6). This bone is preserved from the asterion and the occipitomastoid suture, to the mastoid region, the petrous part, the entire tympanic bone and mandibular fossa, and the near complete sphenosquamosal suture. The surface detail is remarkable. The squamous portion of the temporal bone is preserved intact superior to the damaged root of the zygomatic arch and the external auditory meatus. The squamosal suture is beautifully preserved for a distance 30 mm anterior to the asterion and about 12 mm above the roof of the external auditory meatus.

The barely distinct mastoid tip is well preserved and is already extensively pneumatized by mastoid cellularization, as revealed from micro-CT scans (Fig. S6). The anteroposterior distance between the porion and the mastoid process tip is approximately 12 mm. A slightly rugose area (approximately 5.5 x 11.6 mm in size) is located on the lateral face of the mastoid process and likely corresponds to the bony attachment of the sternocleidomastoid muscle. It is barely inflected inferiorly and its tip is not separated (either by a notch or a groove) from the very shallow digastric fossa located anteriorly and medially. There is no supramastoid crest separated from the mastoid process. When compared to the AL 333-105 *A. afarensis* child, in the absence of supramastoid, occipitomastoid crests and juxtamastoid process, the mastoid process in KW 10840 is much less robust.

The left mandibular fossa is 19 mm broad mediolaterally (15 and 23.8 mm in the *A. africanus* Taung child and the AL 333-105 *A. afarensis* specimen, respectively). Although approximately half of the fossa projects lateral to the external acoustic porus, the mandibular fossa does not project lateral to a sagittal plane through the facies temporalis (in AL 333-105, a large proportion of the mandibular fossa lies lateral to this plane).

The postglenoid is poorly developed and, in a coronal cross section, roughly triangle-shape. Its inferior summit extends about 5.3 mm below the deepest point in the mandibular fossa. A part of the anterior surface of the tympanic bone is articular. Behind the flat preglenoid plane, the coronally concave posterior slope of the articular eminence is low and grades smoothly with the fossa. The entoglenoid process is smooth and delineates partly the foramen spinosum that is splitted by the sphenosquamosal suture.

The external auditory meatus is a large, sub-elliptical in shape (major axis is 12.4 mm long, and its minor short axis is 9.2 mm), with its long axis sloping medially, a nearly vertical anterior wall with its anterior face is separated from the postglenoid process of the mandibular fossa by a distinct groove. The slightly concave inferior face of the tympanic bone is rectangular in shape and 11.7 mm long antero-posteriorly.

The inferolateral edge of the tympanic bone is thin and much recessed medially from the level of the porion, leaving a distinct oval hollow laterally. This morphology is also present in the AL 333-105 *A. afarensis* child and the DNH 34 specimen (see above), and is likely due to the young age of KW 10840. In modern humans, after the closure of the foramen of Huschke, the tympanic plate continues to grow laterally, displacing the lateral edge of this bone laterally (Scheuer and Black 2000).

At the anterior rim of the carotid foramen (circular and about 3.2 mm in diameter), a small and sharp crista petrosa is prolonged laterally into the well-developed vaginal process of the styloid. The crista petrosa arises from the anterolateral rim of the carotid foramen and is well delineated from the base of the Eustachian process (arising topographically from within the squamotympanic fissure) by a distinct and shallow 4.5 mm wide depression. In the *A. afarensis* AL 333-105 specimen, we observe the same relationship as in KW 10840. However, AL 333-105 differs from KW 10840 because the former does not show a vaginal process of the styloid. Moreover, KW 10840 does not show a depression separating the anterior rim of the carotid foramen from the base of the Eustachian process. Unfortunately, the Taung child does not preserve enough details of this morphology.

The long axis of the petrous portion of the left temporal bone deviates from the tympanic axis by approximately a 30° clockwise rotation (in exocranial and inferior view). The plane of the posterior semicircular canal is parallel to the long axis of the petrous and therefore does not approximate the mid-sagittal plane.

Endocranially, the petrous bone is intact. Its endocranial posterior surface (facies posterior partis petrosae) or cerebellous surface shows the pronounced and broad depression of the cerebellar fossa, just supero-lateral to the opening of the vestibular acqueduc. Further lateral is a deeply impressed groove for the sigmoid sinus (5.2 mm wide) lying immediately anterior to the occipitomastoid suture where a small area of the cerebella fossa is also preserved.

**Text S2**

**Measurements of arc lengths, areas, indices and angles of the BL**

We measure the external cochlear length (ECL) and the oval window area (OWA) by following the measurement protocol described in references 3, 45 and 46. We also measure the transverse labyrinthine index (TLI), the inclination of the ampullar line and the cochlear basal turn relative to the orientation of the horizontal (or lateral) semi-circular canal (APA < LSCm and COs < LSCm, respectively), as defined in reference 30. Three curves are digitized on the HSC (between Ld1-Ld2), the PSC (between Ld3-Ld4) and the ASC (between Ld4-Ld5) in order to measure the three corresponding arc lengths (HSCL, PSCL and ASCL, respectively). On the PSC, we measure the arc lengths below and above the plane that best fit the HSC (PSCL.B and PSCL.A, respectively). We also measure six line segments (linear distances) between the following landmarks: (i) Ld3-Ld6 (HELPAM), (ii) Ld4-Ld6 (HELCRS), (iii) Ld5-Ld6 (HELAAM), (iv) Ld3-Ld5 (or “ampullar line”) (PAMAAM), (v) Ld4-Ld3 (joining two extremities of the PSC) (CRSPAM), (vi) Ld4-Ld5 (CRSAAM).

We compute the following 11 indices: (i) the ratio dividing the arc length of the PSC (arc length between Ld3-Ld4) situated below and above the HSC (or Posterior Semi-circular Canal index 1, PSCI1), (ii) the ratio dividing the line segment (linear distance) between Ld3 and the plane best-fitting HSC (HSCP) by the Ld3-Ld4 line segment (PSCI2), (iii) the ECL/HSCL, ECL/PSCL, ECL/ASCL, HSCL/PSCL, HSCL/ASCL, PSCL/ASCL indices, (iv) the HEL1 ratio between HELPAM (Ld3-Ld6) and HELCRS (Ld4-Ld6), (v) the HEL2 ratio between HELPAM (Ld3-Ld6) and HELAAM (Ld5-Ld6), (vi) the CRS2 ratio between CRSPAM (Ld3-Ld4) and PAMAAM (Ld3-Ld5). Finally, we measure the following eight angles: (i) between the Ld2-Ld4 and CRSPAM (Ld4-Ld3) line segments (CRS1), (ii) between the PAMAAM and CRSPAM line segments (CRS3), (iii) between the Ld1-Ld2 and Ld2-Ld4 line segments (inclination of the common crus) (CRS4), (iv) between the Ld3-Ld2 and Ld2-Ld4 line segments (inclination of the common crus) (CRS5), (v) between the Ld2-Ld1 and Ld1-Ld5 line segments (inclination of the anterior ampulla) (AAM1), (vi) ) between the Ld4-Ld1 and Ld1-Ld5 line segments (inclination of the anterior ampulla) (AAM2), (vii) between the Ld1-Ld2 and Ld1-Ld6 line segments (inclination of the cochlea) (CO1), (viii) between the Ld1-Ld5 and the Ld1-Ld6 line segments (inclination of the cochlea) (CO2).

In order to assess measurement errors, each variable was measured twice on all the fossil specimens with more than a one-day interval between each trial. For each BL, the repeats cluster closely together. The distances between the repeats of the same BL are always significantly closer to one another than they are to any other specimen. We also observe a high reproducibility between the repeats (Wilcoxon signed-rand tests, p< 0.01), including for OWA that requires the most careful consideration (Fig. S14).

**
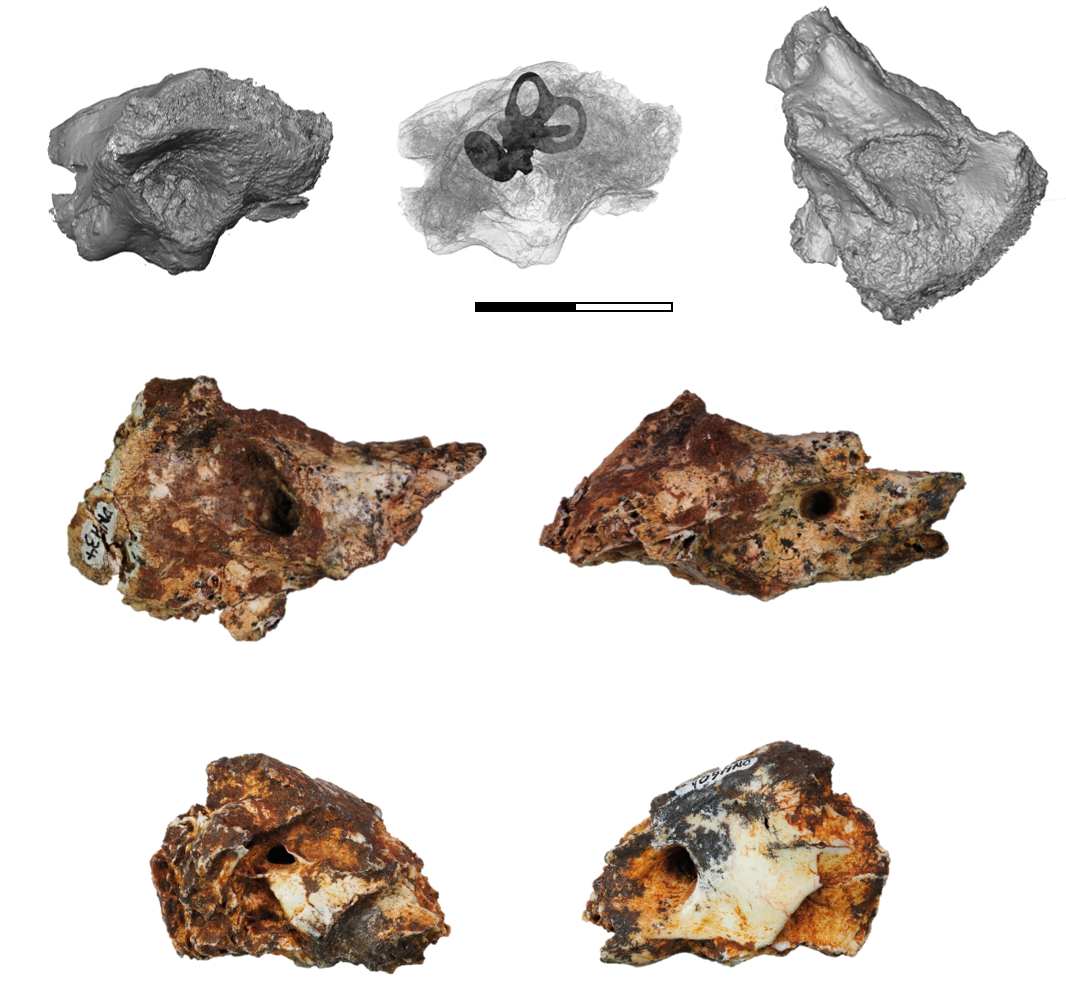
**

**Fig. S1. The DNH 34 and DNH 60 specimens.** From left to right. Top row, Surface reconstructions of the DNH 34 right petrous in lateral (also in transparency to show its bony labyrinth) and inferior views. Middle row, Photographs of the DNH 34 right petrous in lateral and medial views. Bottom row, Photographs of the DNH 60 right petrous in lateral and medial views. Scale: 2 cm.


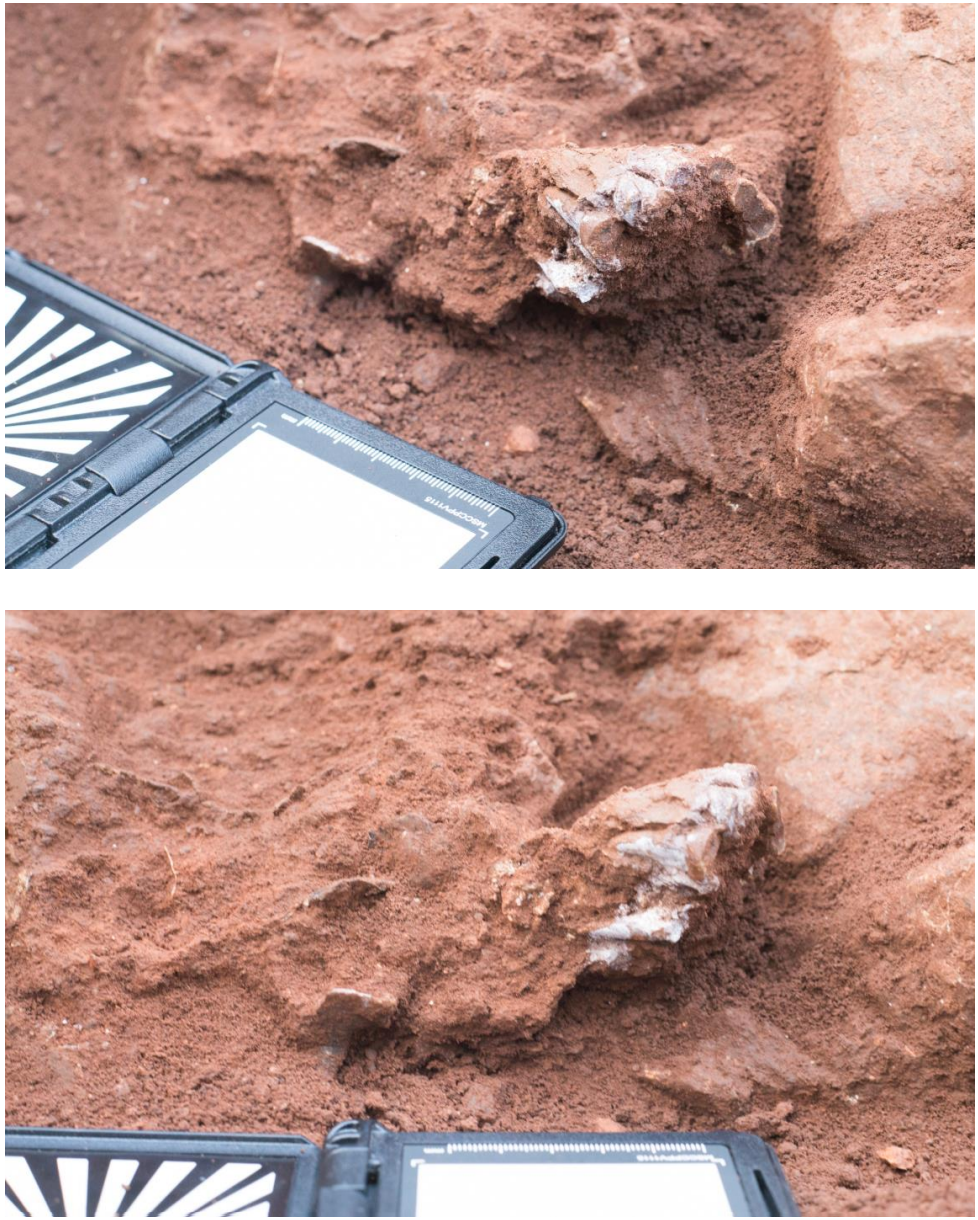


**Fig. S2. The upper maxillary dentition of the KW 9900 adult specimen.** Two photographs taken (by J. Braga) upon the discovery of the KW 9900 dentition in situ within Unit P in October 2016. Top row, Occlusal view. Bottom row, Right lateral view.

**
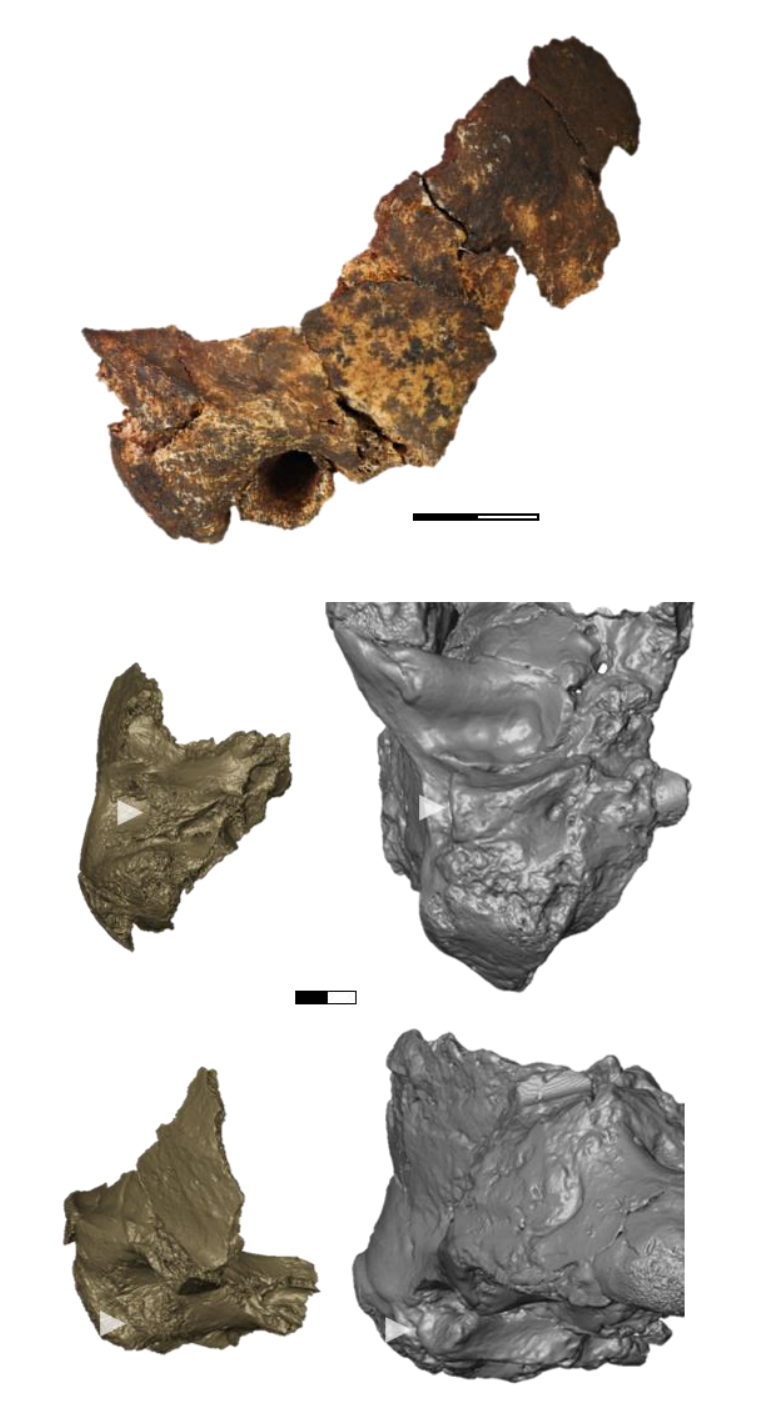
**

**Fig. S3. The larger cranial fragment of the KW 9900 specimen.** Top row, Right temporal bone articulated with a short fragment of the right parietal. Middle and bottom rows, Comparison between the KW 9990 (left) and the TM 1517 (right) right temporal bone shown in inferior (middle row) and anterolateral (bottom row) views. The white arrows show the lateral extension of the tympanic bone.

**
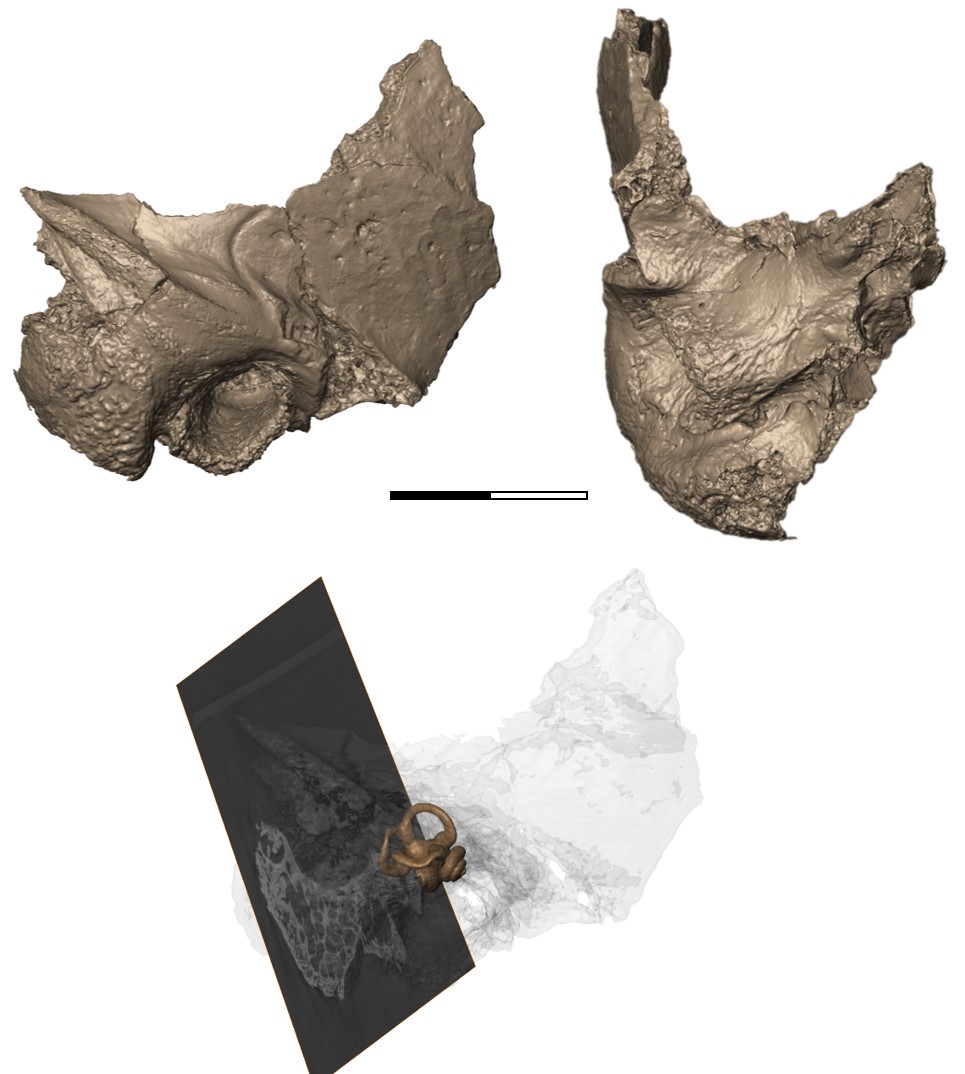
**

**Fig. S4. The right temporal bone of the KW 9900 adult specimen.** Top row, From left to right, Surface reconstructions of the KW 9900 right temporal in lateral (the horizontal semicircular canal of the bony labyrinth is used to define the horizontal plane) and inferior views. Bottom row, Surface reconstruction in transparency of the KW 9900 right temporal in lateral view to show its bony labyrinth. Scale: 2 cm.

**
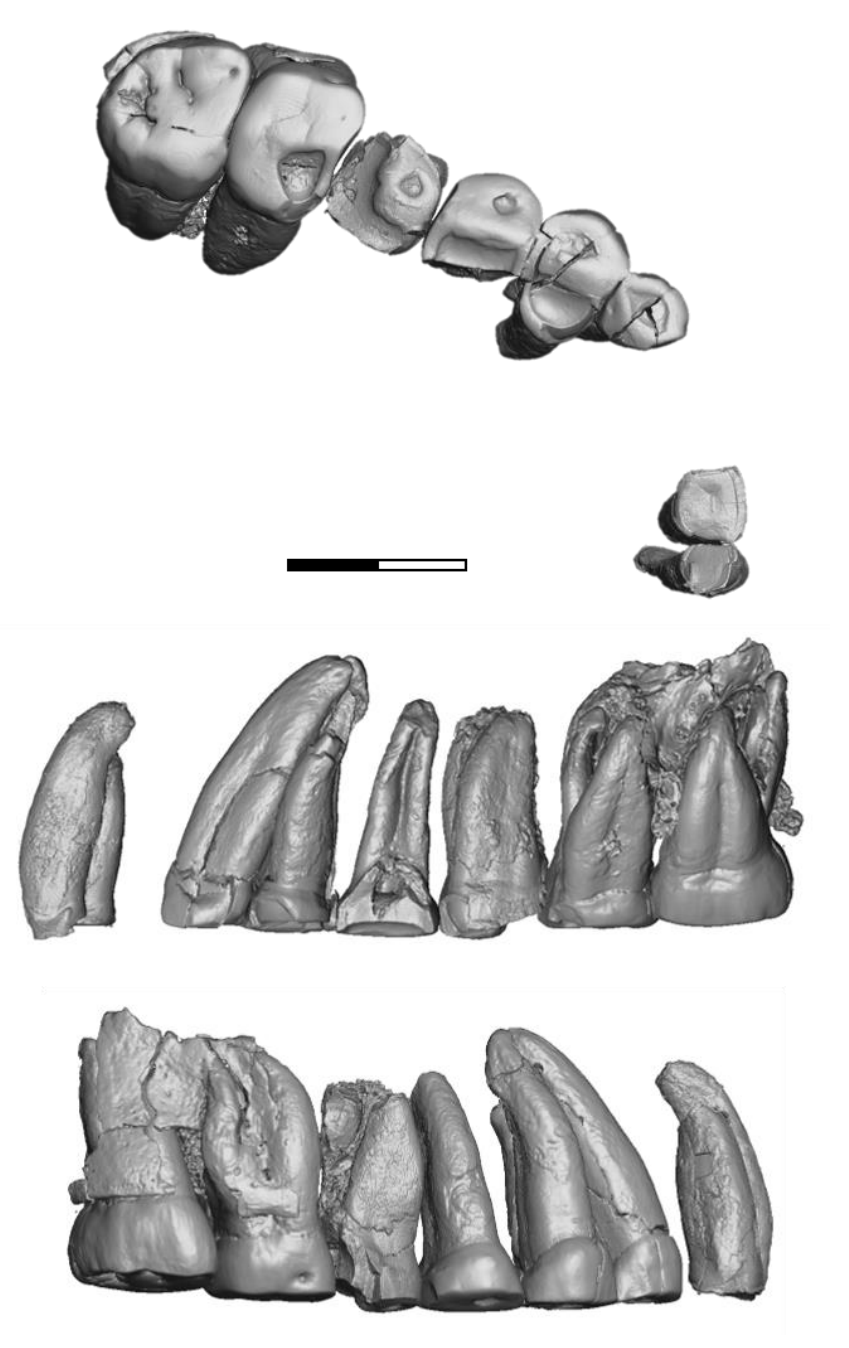
**

**Fig. S5. Maxillary permanent teeth of the KW 9900 adult specimen.** Top row, Surface reconstruction in occlusal view (C-M3, right side, and I1-I2, left side). Middle row, Surface reconstruction in lingual view. Bottom row, Surface reconstruction in buccal view.


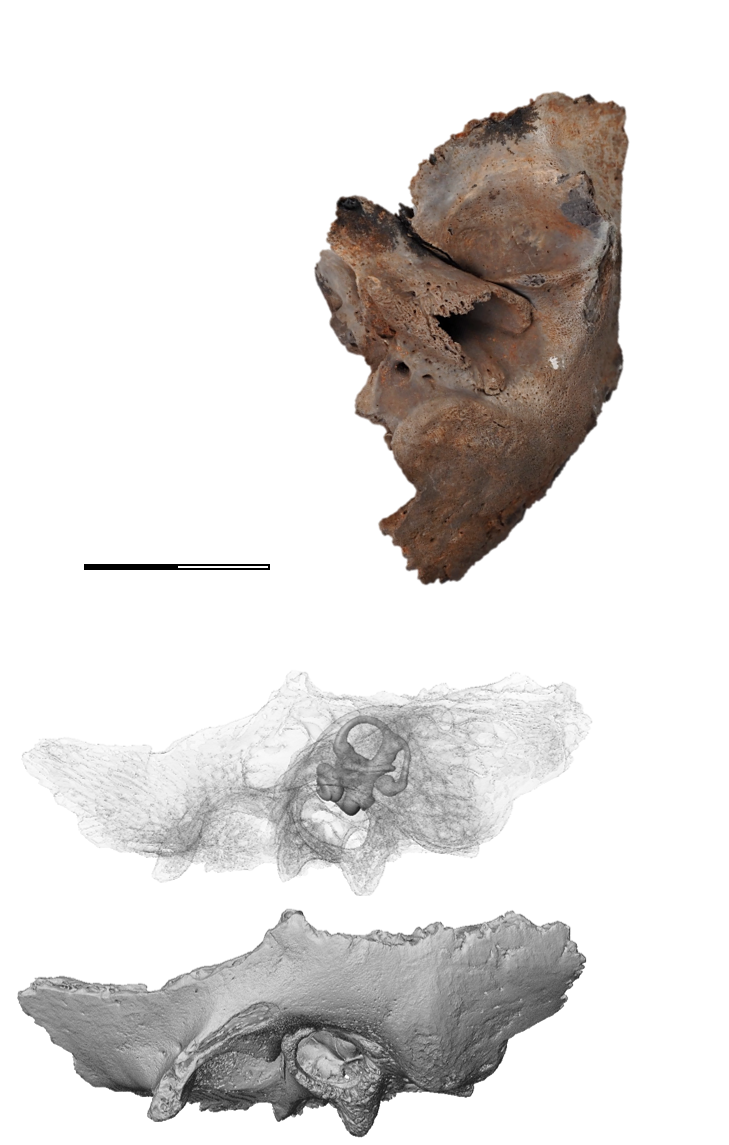


**Fig. S6. The left temporal bone of the KW 10840 infant.** Top row, Photograph of the KW 10840 left temporal in inferior view. Middle and bottom rows, Surface reconstructions of the KW 10840 left temporal in lateral view (also in transparency to show its bony labyrinth, middle). Scale: 2 cm.


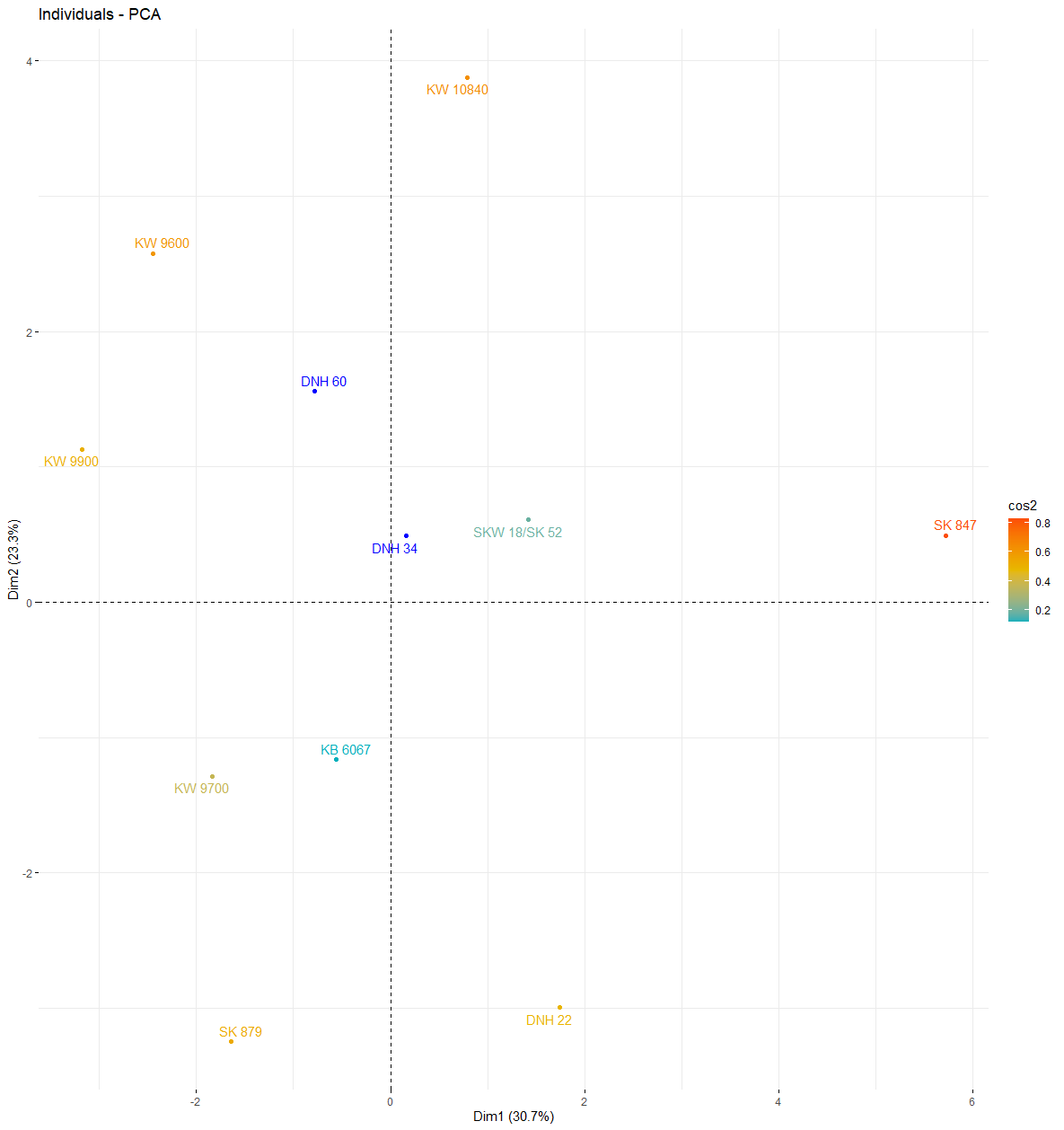


**Fig. S7. Binary plot from a PCA (PC1 versus PC2) of 10 angles and 12 indices of the BL.** The specimens represent *Paranthropus robustus* from the Kromdraai (n=5), Drimolen (n=1) and Swartkrans (n=2) sites, one early *Homo* specimen from Swartkrans (SK 847), and two additional fossils from Drimolen (DNH 34 and DNH 60, in blue) here considered as “indeterminate” and projected onto the statistical space to identify their closest neighbours. For each specimen (except DNH 34 and DNH 60), its “cos2” value (or “squared cosine”) is color-coded indicates the quality of its representation of this PC1 versus PC2 plot.





**Fig. S8. Semi-circular canal (SCC) variation using canonical variate analysis (CVA).** Morphospace obtained showing the first versus the second mode of variation when 7 semilandmarks per SCC are considered (Methods). *P. robustus* from the Kromdraai (n=5, dark blue), Drimolen (n=3, light blue) and Swartkrans (n=2, light blue) sites, *A. africanus* (brown) from the Sterkfontein (n=9), Makapansgat (MLD 31) and Taung (holotype) sites, early *Homo* from Swartkrans (SK 847, red), and modern humans (burgundy color) (n=10 with equal numbers of females and males). Two fossil specimens (StW 151 and Stw 53, black) here considered as “indeterminate” are projected onto the biplots.


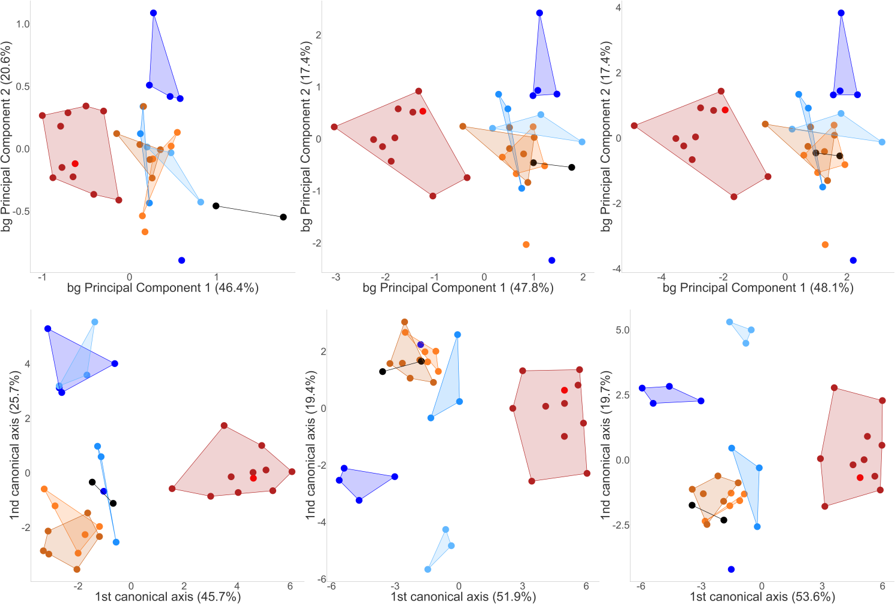


**Fig. S9. Assessments of semi-circular canal (SCC) variation using Procrustes and Mahalanobis distances after a Generalized Procrustes Analysis (GPA)**. Morphospace obtained from either a between-group principal component analysis (bg PCA) (top row) or a canonical variate analysis (CVA) (bottom row) after a GPA showing the first versus the second mode of variation when 1 (left column), 18 (middle column) and 48 (right column) semilandmarks per SCC are considered (Methods). *P. robustus* from the Kromdraai (n=5, dark blue), Drimolen (n=3, light blue) and Swartkrans (n=2, light blue) sites, *A. africanus* (brown) from the Sterkfontein (n=9), Makapansgat (MLD 31) and Taung (holotype) sites, early *Homo* from Swartkrans (SK 847, red), and modern humans (burgundy color) (n=10 with equal numbers of females and males). Two fossil specimens (StW 151 and Stw 53, black) here considered as “indeterminate” are projected onto the biplots.

**
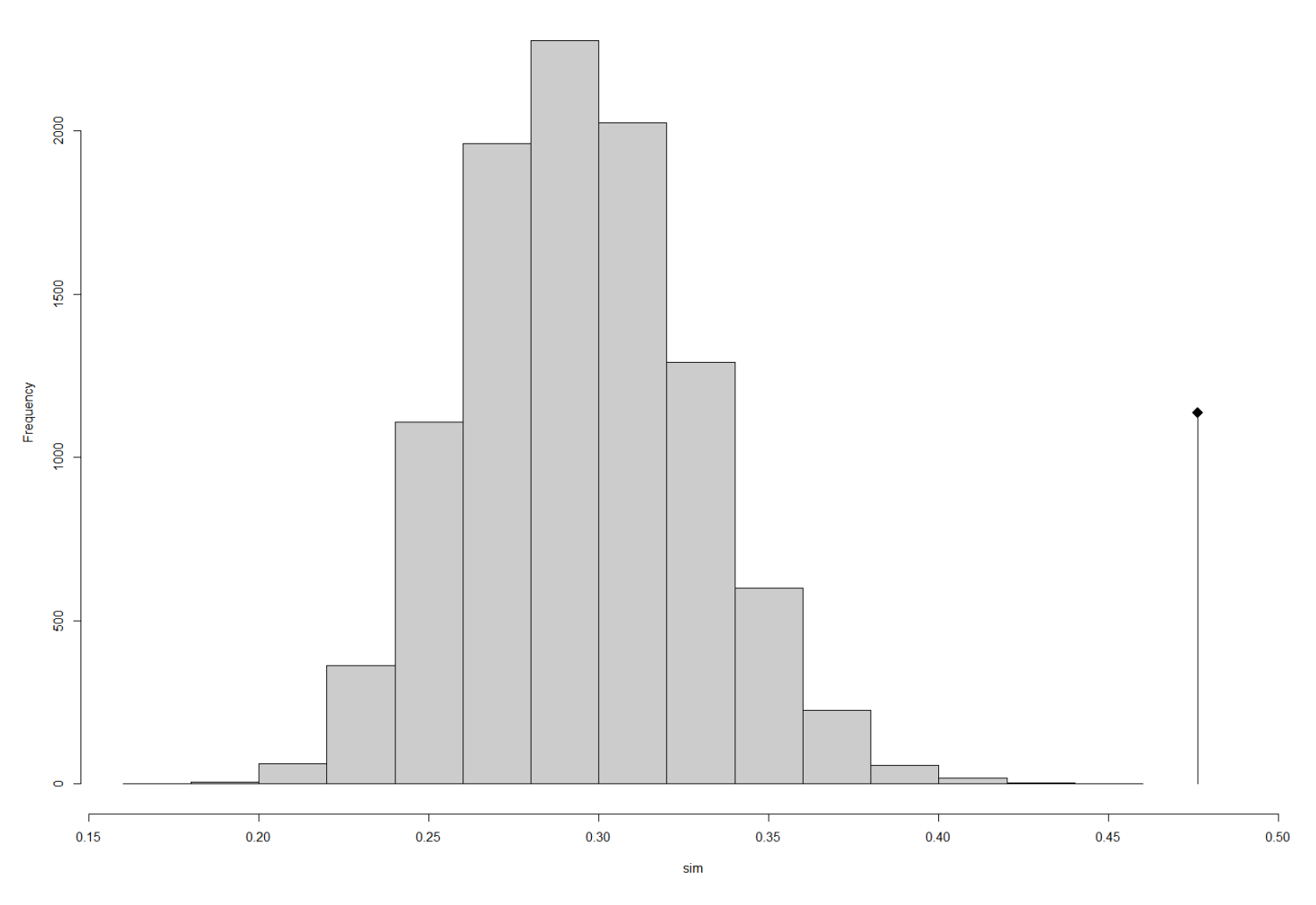
**

**Fig. S10. Histogram of the 999 simulated values of the randomization test to test the statistical significance of the bgPCA presented in Fig. 2a.** The observed value is given by the vertical line, at the right of the histogram. The p-value is highly significant, which confirms the existence of differences between sampling sites. The plot shows that the observed value is very far to the right of the histogram of simulated values.

**

**

**Fig. S11. Assessments of semi-circular canal (SCC) variation in modern species using Procrustes distances after a Generalized Procrustes Analysis (GPA)**. Morphospace obtained from a between-group principal component analysis (bg PCA) showing bgPC1 versus bgPC2 when 7 landmarks per SCC and four modern species are considered: modern humans (n=10, burgundy color), bonobos (n=10, light green), common chimpanzees (n=10, dark green), gorillas (n=10, cyan). The following fossil specimens are projected onto this biplot: *P. robustus* from the Kromdraai (n=5, dark blue), Drimolen (n=3, light blue) and Swartkrans (n=2, light blue) sites, *A. africanus* (brown) from the Sterkfontein (n=9), Makapansgat (MLD 31) and Taung (holotype) sites, early *Homo* from Swartkrans (SK 847, red), and the two fossil specimens StW 151 and Stw 53 considered as “indeterminate”.





**Fig. S12. Assessments of semi-circular canal (SCC) variation after a multidimensional scaling (MDS) from computational anatomy.** Morphospace showing MDS 1 versus MDS 2 with four modern species - modern humans (n=10, burgundy color), bonobos (n=10, light green), common chimpanzees (n=10, dark green), gorillas (n=10, cyan) - *P. robustus* from the Kromdraai (n=5, dark blue), Drimolen (n=3, light blue) and Swartkrans (n=2, light blue) sites, *A. africanus* (brown) from the Sterkfontein (n=9), Makapansgat (MLD 31) and Taung (holotype) sites, early *Homo* from Swartkrans (SK 847, red), and the two fossil specimens StW 151 and Stw 53 considered as “indeterminate”.


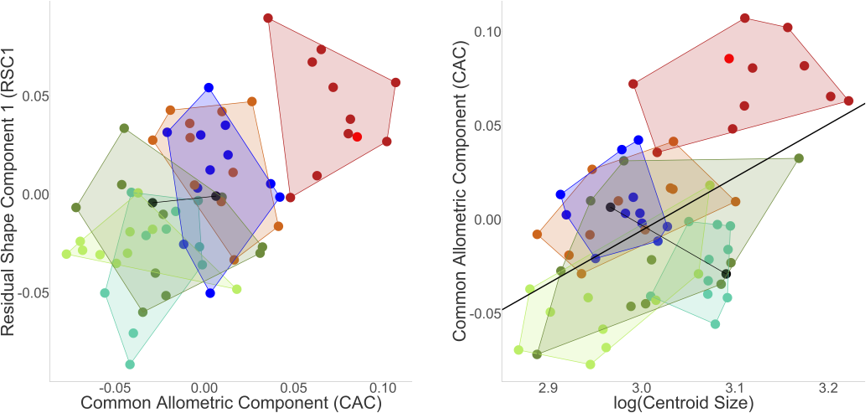


**Fig. S13. Assessments of semi-circular canal (SCC) variation and allometry in modern and fossil species using Procrustes distances after a Generalized Procrustes Analysis (GPA)**. Allometry obtained from a principal component analysis (PCA) showing CAC versus RSC1 scores (left) and log centroid size versus CAC (right) when 7 landmarks per SCC are considered in modern humans (n=10, burgundy color), bonobos (n=10, light green), common chimpanzees (n=10, dark green), gorillas (n=10, cyan), *P. robustus* from the Kromdraai (n=5, dark blue), Drimolen (n=3, light blue) and Swartkrans (n=2, light blue) sites, *A. africanus* (brown) from the Sterkfontein (n=9), Makapansgat (MLD 31) and Taung (holotype) sites, early *Homo* from Swartkrans (SK 847, red), and the two fossil specimens StW 151 and StW 53 considered as “indeterminate”.


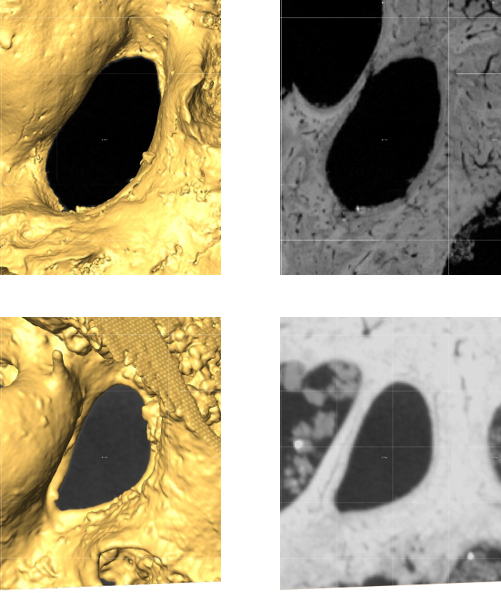


**Fig. S14. Oval window area and middle ear cavity in *P. robustus.*** 3D reconstructions from micro-CT data of the middle ear cavity (left column) and oblique slices used to measure the oval window area (OWA) (right column) in the SK 879 (OWA: 4.3 mm^2^) (top row) and DNH 22 (OWA: 2.7 mm^2^) (bottom row) specimens shown using the same scale.

| **Specimen** | **Sex** | **Institution** | **Micro-CT system** | **Voxel Size** |
| --- | --- | --- | --- | --- |
| *Australopithecus* *africanus* (n=11) |  |  |  |  |
| TAUNG | Unknown | WITS | Nikon Duo | 65.03 |
| MLD 31 | Unknown | WITS | Nikon Duo | 31.9 |
| STW 329 | Unknown | WITS | Nikon Duo | 33.1 |
| STW 498* | Unknown | WITS | Nikon Duo | 28.1 |
| STW 252/255/259* | Unknown | WITS | Nikon Duo | 33.1 |
| STW 98 | Unknown | WITS | Nikon Duo | 33.1 |
| STS 5 | Unknown | DNMNH | Metris X-Tek XT H225L | 76.15 |
| STW 578* | Unknown | WITS | Morphosource (3D Model) |  |
| STW 573* « Little Foot » | Unknown | WITS | Morphosource (3D Model) |  |
| STW 504/505* | Unknown | WITS | Nikon Duo | 30.5 |
| STS 19 | Unknown | DNMNH | Metris X-Tek XT H225L | 62.9 |
| *Paranthropus robustus* (n=11) |  |  |  |  |
| SK 879 | Unknown | DNMNH | Metris X-Tek XT H225L | 9.2 |
| SKW 18 / SK 52 | Unknown | WITS | Metris X-Tek XT H225L | 11.14 |
| SK 83 | Unknown | DNMNH | Metris X-Tek XT H225L | 75.98 |
| KB 6067 | Unknown | DNMNH | Swiss Light Source, TOMCAT | 7.4 |
| KW 9600 | Unknown | WITS | Nikon Duo | 19.8 |
| KW 9700 | Unknown | WITS | Nikon Duo | 25.5 |
| KW 9900 | Unknown | WITS | Nikon Duo | 43.5 |
| KW 10840 | Unknown | WITS | Nikon Duo | 64.1 |
| DNH 22 | Unknown | WITS | Nikon Duo | 26.7 |
| DNH 34 | Unknown | WITS | Nikon Duo | 36.71 |
| DNH 60 | Unknown | WITS | Nikon Duo | 36.71 |
| Early *Homo* (n=1) |  |  |  |  |
| SK 847 | Unknown | DNMNH | Metris X-Tek XT H225L | 21.7 |
| Indeterminate (n=2) |  |  |  |  |
| SW 151 | Unknown | WITS | Nikon Duo | 28.3 |
| StW 53 | Unknown | WITS | Nikon Duo | 30.5 |
| *Gorilla gorilla* (n=10) |  |  |  |  |
| RG 2259 | Unknown | MRAC | RX-solutions EasyTom XL duo | 32.6 |
| MRAC 35274 | Male | MRAC | RX-solutions EasyTom XL duo | 32.6 |
| MRAC 804 | Male | MRAC | RX-solutions EasyTom XL duo | 110 |
| MCZ 37265 | Female | MCZ | [www.MorphoSource.org](http://www.MorphoSource.org) [1] | 11.64 |
| MCZ 49006 | Female | MCZ | [www.MorphoSource.org](http://www.MorphoSource.org) [1] | 12.55 |
| MRAC 29102 | Female | MRAC | RX-solutions EasyTom XL duo | 100 |
| MRAC 31132 | Female | MRAC | RX-solutions EasyTom XL duo | 20.0 |
| MRAC 22763 | Male | MRAC | RX-solutions EasyTom XL duo | 20.0 |
| RG 35274 | Male | MRAC | RX-solutions EasyTom XL duo | 32.6 |
| MCL 1994 | Female | MCL | www. <http://paleo.esrf.eu> [2] | 22.93 |
| *Homo sapiens* (n=10) |  |  |  |  |
| EMBR 121 | Female | IANPS | XtremeCT | 41.0 |
| EMBR 212 | Female | IANPS | XtremeCT | 41.0 |
| EMBR 281 | Female | IANPS | XtremeCT | 41.0 |
| EMBR 136 | Male | IANPS | XtremeCT | 41.0 |
| EMBR 179 | Male | IANPS | XtremeCT | 41.0 |
| EMBR 205 | Male | IANPS | XtremeCT | 41.0 |
| EMBR 383 | Male | IANPS | XtremeCT | 41.0 |
| EMBR 384 | Female | IANPS | XtremeCT | 41.0 |
| EMBR 473 | Male | IANPS | XtremeCT | 41.0 |
| EMBR 479 | Male | IANPS | XtremeCT | 41.0 |
| *Pan paniscus* (n=10) |  |  |  |  |
| MRAC 29007 | Male | MRAC | XtremeCT | 41.0 |
| MRAC 84035M6 | Unknown | MRAC | XtremeCT | 41.0 |
| MRAC 27000 | Male | MRAC | RX-solutions EasyTom XL duo | 47.45 |
| MRAC 84036M7 | Unknown | MRAC | RX-solutions EasyTom XL duo | 48.70 |
| **Specimen** | **Sex** | **Institution** | **Micro-CT system** | **Voxel Size** |
| MRAC 27014 | Female | MRAC | RX-solutions EasyTom XL duo | 43.69 |
| MRAC 29003 | Female | MRAC | XtremeCT | 41.0 |
| MRAC 29051 | Male | MRAC | XtremeCT | 41.0 |
| MRAC 15294 | Male | MRAC | RX-solutions EasyTom XL duo | 20.0 |
| MRAC 15295 | Male | MRAC | RX-solutions EasyTom XL duo | 20.0 |
| MRAC 15296 | Female | MRAC | RX-solutions EasyTom XL duo | 20.0 |
| *Pan troglodytes* (n=10) |  |  |  |  |
| MCL 1754 | Male | MCL | www. <http://paleo.esrf.eu> [2] | 22.93 |
| MCL 1799 | Male | MCL | www. <http://paleo.esrf.eu> [2] | 22.93 |
| MCZ 46414 | Female | MCZ | [www.MorphoSource.org](http://www.MorphoSource.org) [1] | 82.2 |
| MRAC 2297 | Male | MRAC | RX-solutions EasyTom XL duo | 20.0 |
| MRAC 31490 | Female | MRAC | RX-solutions EasyTom XL duo | 85.0 |
| MRAC 80 | Female | MRAC | RX-solutions EasyTom XL duo | 65.0 |
| MRAC 31547 | Female | MRAC | RX-solutions EasyTom XL duo | 75.0 |
| MRAC 82032M8 | Unknown | MRAC | RX-solutions EasyTom XL duo | 84.0 |
| MRAC 15976 | Male | MRAC | RX-solutions EasyTom XL duo | 20.0 |
| MRAC 84036M9 | Unknown | MRAC | RX-solutions EasyTom XL duo | 65.0 |

**Table S1**. List of specimens investigated in this study. Specimens marked with* have been interpreted as representing a « second, larger-toothed species » distinct from *A. africanus* on the basis of dental and facial morphological features^7^. Institutions are: DNMNH (Ditsong National Museum of Natural History, Pretoria, South Africa; formerly Transvaal Museum), IANPS (Institut d’Anatomie Normale et Pathologique de Strasbourg, France), MHNT (Muséum d’Histoire Naturelle de Toulouse, France), MRCA (Musée Royal de l’Afrique Centrale, Tervuren, Belgium), MCL (Musée des Confluences de Lyon), MCZ (Museum of Comparative Zoology, Harvard), UT-PS (Université de Toulouse, Paul Sabatier, France), WITS (Evolutionary Studies Institute, University of the Witwatersrand, Johannesburg, South Africa). Voxel size is indicated in microns. Notes: [1] The files were downloaded from www.MorphoSource.org, Duke University. Lynn Lucas and Lynn Copes provided access to these data. The reference of the original article linked to the data is: Copes, L.E. & Kimbel, W.H. Cranial vault thickness in primates: *Homo erectus* does not have uniquely thick vault bones. J. Hum. Evol. 90, 120-134 (2016). The collection of these data was funded by NSF DDIG #0925793, and a Wenner-Gren Foundation Dissertation Grant #8102 (both to Lynn Copes). [2] The files were downloaded from the ESRF heritage database for palaeontology, evolutionary biology and archaeology; www. http://paleo.esrf.eu/. The reference of the original article linked to the data is: Nengo, I. *et al*. New infant cranium from the African Miocene sheds light on ape evolution. Nature, 548:169-174 (2017).

|  | **Swartkrans** | | | |  | **Drimolen** | | | |  | **KW 9900** | |
| --- | --- | --- | --- | --- | --- | --- | --- | --- | --- | --- | --- | --- |
|  | **n** | **Mean** | **Range** | **SD** |  | **n** | **Mean** | **Range** | **SD** |  | **Right** | **Left** |
| I1 MD | 16 | 8.9 | 7.0-9.8 | 0.7 |  | 4 | 8.5 | 8.0-8.9 | 0.4 |  | - | - |
| I1 BL | 15 | 7.3 | 6.3-8.1 | 0.5 |  | 3 | 6.8 | 6.5-7.1 | 0.3 |  | 7.0 | 7.2 |
| I2 MD | 9 | 6.5 | 5.9-8.1 | 0.7 |  | 3 | 5.7 | 5.0-6.3 | 0.7 |  | - | 6.2 |
| I2 BL | 9 | 6.6 | 5.8-7.9 | 0.6 |  | 3 | 6.1 | 5.4-7.0 | 0.8 |  | 6.0 | - |
| C MD | 19 | 8.5 | 7.4-9.7 | 0.6 |  | 6 | 8.7 | 7.9-9.9 | 0.7 |  | - | 7.9 |
| C BL | 19 | 9.3 | 8.4-11.1 | 0.7 |  | 8 | 8.8 | 7.9-9.9 | 0.8 |  | 8.5 | 8.7 |
| P3 MD | 19 | 9.9 | 9.2-11.1 | 0.5 |  | 4 | 9.2 | 9.0-9.7 | 0.3 |  | - | 9.5 |
| P3 BL | 15 | 14.2 | 12.9-15.3 | 0.7 |  | 3 | 13 | 12.8-13.4 | 0.3 |  | - | 13.4 |
| P4 MD | 22 | 10.6 | 9.4-12.1 | 0.6 |  | 4 | 9.8 | 9.2-10.1 | 0.4 |  | 9.0 | - |
| P4 BL | 20 | 15.0 | 13.6-16.5 | 0.8 |  | 4 | 13.9 | 13.6-14.3 | 0.3 |  | - | 14.8 |
| M1 MD | 20 | 13.3 | 12.0-15.0 | 0.7 |  | 5 | 12.8 | 12.0-14.1 | 0.8 |  | - | - |
| M1 BL | 19 | 14.9 | 14.4-16.8 | 0.6 |  | 5 | 14.2 | 13.6-15.2 | 0.7 |  | - | - |
| M2 MD | 20 | 14.1 | 12.8-15.7 | 0.9 |  | 5 | 13.1 | 11.6-15 | 1.3 |  | (11.6) | (12.4) |
| M2 BL | 20 | 15.9 | 14.3-17.0 | 0.8 |  | 5 | 14.7 | 14.0-16.0 | 0.8 |  | 15.6 | 15.3 |
| M3 MD | 16 | 14.9 | 12.7-17.2 | 1.1 |  | 7 | 13.7 | 12.1-14.8 | 0.9 |  | (13.4) | (14.7) |
| M3 BL | 16 | 17.0 | 15.9-18.2 | 0.6 |  | 7 | 15.3 | 14.2-16.4 | 0.8 |  | 15.5 | 15.9 |

**Table S2.** Measurements of the KW 9900 permanent maxillary teeth and descriptive statistics (taken from reference 17) of the Swartkrans (SK) and Drimolen (DR) *P. robustus* samples. Measurements are in mm. Estimated values in parentheses. MD, mesiodistal; BL, buccolingual.

| **Abbreviation** | **Measure** | **Name** | **Definition** |
| --- | --- | --- | --- |
| ECL | Arc length | External cochlear length | References 15, 23 |
| OWA | Area | Oval window area | References 15, 22, 23 |
| TLI | Index | Transverse labyrinthine index | Reference 12, Spoor, 1993 |
| HZCO | Angle | Angle between the ampular line and the HSC | Reference 12, Spoor, 1993 |
| HZAPA | Angle | Angle between the cochlear basal and the HSC | Reference 12, Spoor, 1993 |
| HSCL | Arc length | Arc length of the horizontal semi-circular canal (between Ld1 and Ld2) | This study |
| PO.B | Arc length | HSCL measured below the HSC | This study |
| PO.A | Arc length | HSCL measured above the HSC | This study |
| PSCL | Arc length | Arc length of the posterior semi-circular canal (between Ld3 and Ld4) | This study |
| PSCI1 | Index | Posterior Semi-circular Canal index 1 | This study |
| PSCI2 | Index | Ratio dividing (i) the line segment between Ld3 and HSCP by (ii) the line segment between Ld3 and Ld4 | This study |
| ASCL | Arc length | Arc length of the anterior semi-circular canal (between landmarks 4 and 5, Ld4-Ld5) | This study |
| HELPAM | Line segment | Linear distance between landmarks 3 and 6 (Ld3-Ld6) | This study |
| HELCRS | Line segment | Linear distance between landmarks 4 and 6 (Ld4-Ld6) | This study |
| HEL1 | Index | Ratio dividing HELPAM and HELCRS (see above) | This study |
| HELAAM | Line segment | Linear distance between landmarks 5 and 6 (Ld5-Ld6) | This study |
| HEL2 | Index | Ratio dividing HELPAM and HELAAM (see above) | This study |
| PAMAAM | Line segment | Linear distance between landmarks 3 and 5 (Ld3-Ld5) | This study |
| CRSPAM | Line segment | Linear distance between landmarks 4 and 3 (Ld4-Ld3) | This study |
| CRSAAM | Line segment | Linear distance between landmarks 4 and 5 (Ld4-Ld5) | This study |
| CRS1 | Angle | Angle between the Ld2-Ld4 and CRSPAM (Ld4-Ld3) line segments (see above) | This study |
| CRS2 | Index | Ratio between CRSPAM and PAMAAM (see above) | This study |
| ECL/HSCL | Index | Ratio dividing ECL and HSCL (see above) | This study |
| ECL/PSCL | Index | Ratio dividing ECL and PSCL (see above) | This study |
| ECL/ASCL | Index | Ratio dividing ECL and ASCL (see above) | This study |
| HSCL/PSCL | Index | Ratio dividing HSCL and PSCL (see above) | This study |
| HSCL/ASCL | Index | Ratio dividing HSCL and ASCL (see above) | This study |
| PSCL/ASCL | Index | Ratio dividing PSCL and ASCL (see above) | This study |
| CRS3 | Angle | Angle between the PAMAAM (Ld3-Ld5) and CRSPAM (Ld4-Ld3) line segments (see above) | This study |
| CRS4 | Angle | Angle between the Ld1-Ld2 and Ld2-Ld4 line segments (inclination of the common crus) (see above) | This study |
| CRS5 | Angle | Angle between the Ld3-Ld2 and Ld2-Ld4 line segments (inclination of the common crus) (see above) | This study |
| AAM1 | Angle | Angle between the Ld2-Ld1 and Ld1-Ld5 line segments (inclination of the anterior ampulla) (see above) | This study |
| AAM2 | Angle | Angle between the Ld4-Ld1 and Ld1-Ld5 line segments (inclination of the anterior ampulla) (see above) | This study |
| CO1 | Angle | Angle between the Ld1-Ld2 and Ld1-Ld6 line segments (inclination of the cochlea) (see above) | This study |
| CO2 | Angle | Angle between the Ld1-Ld5 and the Ld1-Ld6 line segments (inclination of the cochlea) (see above) | This study |

**Table S3.** List of the 35 variables used in this study. They include one area, 6 arc lengths, 6 linear distances (line segments), 12 indices and 10 angles. The landmarks (Ld) used to define the lengths, distances, indices and angles are defined in the Methods and illustrated in Fig. 1. Abbreviations: Ld, landmark; HSCP, plane best-fitting HSC (HSCP); ASCP, plane best-fitting HSC (ASCP).

|  | Aa | Pr SK | *Homo* | Pr KW P | Pr KB QP | Pr SK | Indet. |
| --- | --- | --- | --- | --- | --- | --- | --- |
| *A. africanus* [Aa] | 11 | 0 | 0 | 0 | 0 | 0 | 0 |
| *P. robustus* SK [Pr SK] | 1 | 2 | 0 | 0 | 0 | 0 | 0 |
| *Homo* | 0 | 0 | 11 | 0 | 0 | 0 | 0 |
| *P. robustus* KW P [Pr KW P] | 0 | 0 | 0 | 4 | 0 | 0 | 0 |
| *P. robustus* KW P [Pr KB QP] | 0 | 0 | 0 | 0 | 1 | 0 | 0 |
| *P. robustus* SK [Pr SK] | 0 | 0 | 0 | 0 | 0 | 3 | 0 |
| Indeterminate [Indet.] | 0 | 0 | 0 | 0 | 0 | 0 | 2 |

**Table S4**. Classifications (with results in frequencies) obtained from a canonical variate analysis and permutation tests (100 rounds) between groups.

| **Group 1** | **Group2** | **p. value** | |
| --- | --- | --- | --- |
|  |  | **CS (SCC)** | **OWA** |
| early *Homo* | *P. robustus* KW&DNH | 0.22 | 0.329 |
| early *Homo* | *P. robustus* SK | 0.67 | 0.667 |
| early *Homo* | *A. africanus* | 0.33 | 0.190 |
| early *Homo* | Indeterminate | 0.67 | 1.000 |
| *P. robustus* KW&DNH | *P. robustus* SK | 0.71 | 0.049* |
| *P. robustus* KW&DNH | *A. africanus* | 0.66 | 0.395 |
| *P. robustus* KW&DNH | Indeterminate | 0.53 | 0.694 |
| *P. robustus* SK | *A. africanus* | 0.77 | 0.059 |
| *P. robustus* SK | Indeterminate | 1.00 | 0.333 |
| *A. africanus* | Indeterminate | 0.51 | 0.390 |
| *A. africanus* STS | *A. africanus** STS | 0.66 | 0.81 |
| *A. africanus* STS | early *Homo* | 0.29 | 0.35 |
| *A. africanus* STS | *P. robustus* DNH | 0.55 | 0.38 |
| *A. africanus* STS | *P. robustus* KW P | 0.35 | 0.47 |
| *A. africanus* STS | *P. robustus* KB QR | 0.86 | 1.00 |
| *A. africanus* STS | *P. robustus* SK | 0.64 | 0.14 |
| *A. africanus* STS | Indeterminate | 0.43 | 0.37 |
| *A. africanus** STS | early *Homo* | 0.67 | 0.50 |
| *A. africanus** STS | *P. robustus* DNH | 1.00 | 0.82 |
| *A. africanus** STS | *P. robustus* KW P | 0.41 | 1.00 |
| *A. africanus** STS | *P. robustus* KB QR | 1.00 | 1.00 |
| *A. africanus** STS | *P. robustus* SK | 1.00 | 0.20 |
| *A. africanus** STS | Indeterminate | 0.86 | 0.80 |
| early *Homo* | *P. robustus* DNH | 0.50 | 1.00 |
| early *Homo* | *P. robustus* KW P | 0.40 | 0.28 |
| early *Homo* | *P. robustus* KB QR | 1.00 | 1.00 |
| early *Homo* | *P. robustus* SK | 0.67 | 0.67 |
| early *Homo* | Indeterminate | 0.67 | 1.00 |
| *P. robustus* DNH | *P. robustus* KW P | 0.23 | 0.59 |
| *P. robustus* DNH | *P. robustus* KB QR | 1.00 | 1.00 |
| *P. robustus* DNH | *P. robustus* SK | 0.80 | 0.20 |
| *P. robustus* DNH | Indeterminate | 1.00 | 1.00 |
| *P. robustus* KW P | *P. robustus* KB QR | 0.80 | 1.00 |
| *P. robustus* KW P | *P. robustus* SK | 0.27 | 0.10 |
| *P. robustus* KW P | Indeterminate | 0.27 | 0.48 |
| *P. robustus* KB QR | *P. robustus* SK | 1.00 | 0.67 |
| *P. robustus* KB QR | Indeterminate | 1.00 | 1.00 |
| *P. robustus* SK | Indeterminate | 1.00 | 0.33 |
| *P.paniscus* | *P.troglodytes* | 0.13038 | 0.00431** |
| *P.paniscus* | *G.gorilla* | 0.00033*** | 0.00061*** |
| *P.paniscus* | *H.sapiens* | 0.00018*** | 0.00043*** |
| *P.troglodytes* | *G.gorilla* | 0.09272 | 0.00061*** |
| *P.troglodytes* | *H.sapiens* | 0.00622** | 0.00043*** |
| *G.gorilla* | *H.sapiens* | 0.02202* | 0.09930 |
| *G.gorilla* F | *G.gorilla* M | 0.2857 | 0.3853 |
| *G.gorilla* F | *H.sapiens* F | 0.1905 | 0.1333 |
| *G.gorilla* F | *H.sapiens* M | 0.0823 | 0.9266 |
| *G.gorilla* F | *P.paniscus* F | 0.0357* | 0.0357* |
| *G.gorilla* F | *P.paniscus* M | 0.0317* | 0.0079** |
| *G.gorilla* F | *P.troglodytes* F | 0.0159* | 0.0195* |
| *G.gorilla* F | *P.troglodytes* M | 1.0000 | 0.0159* |
| *G.gorilla* M | *H.sapiens* F | 0.3429 | 0.0396* |
| *G.gorilla* M | *H.sapiens* M | 0.1143 | 0.2835 |
| *G.gorilla* M | *P.paniscus* F | 0.0571 | 0.0497* |
| *G.gorilla* M | *P.paniscus* M | 0.0159* | 0.0195* |
| *G.gorilla* M | *P.troglodytes* F | 0.0286* | 0.0284* |
| *G.gorilla* M | *P.troglodytes* M | 1.0000 | 0.0294* |
| *H.sapiens* F | *H.sapiens* M | 0.2571 | 0.1055 |
| *H.sapiens* F | *P.paniscus* F | 0.0571 | 0.0497* |
| *H.sapiens* F | *P.paniscus* M | 0.0317* | 0.0195* |
| *H.sapiens* F | *P.troglodytes* F | 0.0286* | 0.0284* |
| *H.sapiens* F | *P.troglodytes* M | 0.4857 | 0.0294* |
| *H.sapiens* M | *P.paniscus* F | 0.0238* | 0.0238* |
| *H.sapiens* M | *P.paniscus* M | 0.0087** | 0.0043** |
| *H.sapiens* M | *P.troglodytes* F | 0.0190* | 0.0139* |
| *H.sapiens* M | *P.troglodytes* M | 0.1143 | 0.0095** |
| *P.paniscus* F | *P.paniscus* M | 0.7857 | 0.6488 |
| *P.paniscus* F | *P.troglodytes* F | 0.2286 | 0.0497* |
| *P.paniscus* F | P *P.troglodytes* M | 0.6286 | 0.1143 |
| *P.paniscus* M | *P.troglodytes* F | 0.4127 | 0.0342* |
| *P.paniscus* M | *P.troglodytes* M | 0.2857 | 0.0851 |
| *P.troglodytes* F | *P.troglodytes* M | 0.8857 | 0.7702 |

**Table S5**. Wilcoxon tests with significant level (*, at 5%, ** at 1%, *** at 0.1%). F, female; M, Male; STS, Sterkfontein DNH, Drimolen; KW&DNH, Kromdraai and Drimolen; KW P, Kromdraai Unit P; KB QR, Kromdraai Unit Q-R; SK, Swartkrans

**Supplementary References**

Braga, J. Ossification du processus styloïde chez les Pongidés. *C. R. Acad. Sci. Paris*, *série II*, **317**, 273-277 (1993).

Dean, M. C. & Wood, B. A. Basicranial Anatomy of Plio-Pleistocene Hominids from East and South Africa. *Am. J. Phys. Anthropol.*  **59**, 53-71 (1982).

Fazekas, I.G. & Kosa, F. Forensic Foetal Osteology (Budapest, Akademiai Kiado, 1978).

Scheuer, L. & Black, S. Developmental Juvenile Osteology (San Diego, CA, Elsevier Academic Press, 2000).

Spoor, F. The comparative morphology and phylogeny of the human bony labyrinth. Ph.D. Thesis (Utrecht University, 1993).

Wood, B. Koobi Fora research project. Volume 4: Hominid cranial remains (Clarendon Press, 1991).
